# Effect of PGPB-enriched organic fertilizer ORGAON^®^PK on the rhizospheric microbiota and biomass of *Lupinus albus* (L.): a sustainable alternative to chemical fertilizer

**DOI:** 10.1186/s40793-025-00827-x

**Published:** 2025-12-07

**Authors:** Marina Robas-Mora, Vanesa Mercedes Fernández-Pastrana, Daniel González-Reguero, Agustín Probanza, Pedro Antonio Jiménez-Gómez

**Affiliations:** Department of Health and Pharmaceutical Sciences, CEU San Pablo University, Montepríncipe Campus, Ctra. Boadilla del Monte Km 5.300, Boadilla del Monte, 28668 Madrid, Spain

**Keywords:** Bacillus pretiosus, Pseudomonas agronomica, Soil microbiome, Metabolic diversity, Antibiotic resistance reduction, Organic waste valorization, Amplicon sequencing (16S rRNA gene), ANCOM-BC analysis

## Abstract

The intensive use of agrochemicals is essential to maintain crop yields, but it has led to overexploitation of land and environmental deterioration. To promote more sustainable agriculture, this study evaluates the novel effects of biofertilizers enriched with plant growth promoting bacteria, such as *Bacillus pretiosus* and *Pseudomonas agronomica*, on *Lupinus albus* var. Orden Dorado, to improve the rhizospheric soil health and plant biomass as well as reducing dependence on chemical fertilizers. The organic matrix ORGAON^®^PK and its sterilized version, both derived from horticultural waste, were tested compared with a traditional chemical fertilizer and a water control. After three months of treatment, metagenomic analyses (16 S rRNA gene amplicons) indicated that the strains remained in the rhizosphere, increasing metabolic diversity without altering the microbial structure (Shannon index). In addition, a significant reduction in the minimum inhibitory concentration against clinical antibiotics (*p* < 0.05) was observed, highlighting the potential of biofertilizers to decrease microbial resistance in the soil. Principal component analysis showed clear differences between treated and control groups, and ANCOM-BC revealed changes in non-culturable bacteria. Biometric analyses revealed increases of 70–88% in shoot weight, ~ 80% in total biomass, and up to 36% in shoot elongation compared with the control. Biofertilizers improved nutritional quality and plant biomass, suggesting their potential as a sustainable and efficient alternative to the use of chemical fertilizers.

**Supplementary Information:**

The online version contains supplementary material available at 10.1186/s40793-025-00827-x.

## Background

Conventional agricultural practices, aimed at high yields, have led to biodiversity loss, soil degradation, and environmental pollution [1], underscoring the urgency of transitioning to systems that balance productivity with environmental conservation [2]. In practice, conventional yields are typically sustained by mineral fertilization (N–P–K, especially synthetic N), chemical control of weeds, pests, and diseases (herbicides, insecticides, fungicides), intensive tillage, and short rotations/monoculture. These practices are associated with declines in pollinators, reductions in soil microbiota, and copper accumulation. In addition, the intensification of synthetic fertilizer and pesticide use tends to reduce soil microbial diversity and soil organic carbon, which lowers water retention and drought resilience [3, 4].

Agricultural production faces critical challenges such as soil degradation, biotic and abiotic stress, and climate change, while contributing significantly as well to global greenhouse gas (GHG) emissions [5, 6]. By 2050, meeting global food demand will require a 60% increase in output [7]. Conventional agricultural practices, focused on maximizing yields, have caused biodiversity loss, environmental pollution and further soil deterioration [5, 8]. Therefore, the search for more sustainable approaches that balance productivity and environmental conservation has intensified [2].

To address this crisis, the UN and FAO promote the transition to a more sustainable agriculture that improves environmental protection and optimizes resources [7]. Recently, the importance of soil microbiota in soil health and in essential processes such as nutrient cycling has been recognized, where metagenomics has proven to be a crucial tool for understanding these complex microbial ecosystems [9, 10].

The recovery of waste to produce organic fertilizers and biofertilizers is key to building a more sustainable agricultural system. Organic fertilizers, derived from natural materials, and biofertilizers, which contain live microorganisms, enhance soil quality while reducing the environmental impact of chemical inputs [11–13]. Plant growth-promoting bacteria (PGPB), commonly applied in biofertilizers, increase nutrient availability, strengthen plant defences, and promote soil health. For instance, certain *Bacillus* strains (e.g., *B. aryabhattai* H19-1, *B. mesonae* H_2_0-5) confer salinity tolerance, while others such as *Pseudomonas spp.* produce ACC deaminase. Other examples include *Halomonas variabilis* HT1 and *Planococcus rifietoensis* RT4, which synthesize exopolysaccharides under saline stress, and *Bacillus thuringiensis* GDB-1, which enhances heavy-metal removal. Moreover, strains like *Proteus mirabilis* T2Cr and CrP450 can mitigate chromium toxicity. Several *Bacillus* and *Pseudomonas* isolates have also been shown to promote hyperaccumulation in plants [14–17]. Notably, these bacteria may help reduce the spread of antibiotic resistance genes in soil, an emerging challenge for agricultural sustainability [18–20].

Antimicrobial resistance (AMR) is one of the leading threats to global health, projected to cause up to 10 million deaths annually and losses of 100 trillion USD in global GDP by 2050. As a fundamental ecosystem, soil acts as a natural reservoir of antibiotic resistance genes (ARGs) and occupies a central place within the One Health framework. However, not all ARGs pose the same risk: only those considered “high-risk” by virtue of their mobility, association with human bacteria, and clinical relevance, represent a direct threat to public health [21]. In this context, plant growth-promoting bacteria (PGPB), particularly *Bacillus* and *Pseudomonas*, offer advantages by providing biocontrol via nonribosomal peptides (e.g., iturin, surfactin, fengycin) and by inducing plant defenses, thereby reducing the need for antimicrobials and lowering the selection pressure that drives the spread of antibiotic resistance genes (ARGs) in agroecosystems. Nevertheless, some strains may carry mobile ARGs or virulence factors, and co-selection by metals/antibiotics from organic amendments can sustain or increase the resistome. Therefore, selecting inoculants free of clinically relevant ARGs and evaluating them under a One Health approach is recommended [22]. Accordingly, this study tests the hypothesis that a residue-derived biofertilizer (ORGAON^®^PK, OPK), particularly when fortified with PGPB (*Bacillus pretiosus*, C1; *Pseudomonas agronomica*, C2), improves *Lupinus albus* performance and reduces community-level MICs in the rhizosphere relative to mineral fertilization and water control.

*Lupinus albus* is characterized by its low requirement for mineral fertilization, due to nitrogen fixation and the development of cluster roots highly efficient in phosphorus uptake, which benefits subsequent crops such as wheat or rapeseed in rotation systems. Historically, it has also been used as a green manure and for erosion control in poor, sandy soils, reinforcing its role in 2–3-year crop rotations rather than as an intercrop. In terms of yield, seed productions of up to 5.1 t/ha and protein productions of 1.9 t/ha have been reported, although with high variability between years and environments. However, it faces significant agronomic challenges such as anthracnose and high alkaloid content, which have limited its competitiveness. Moreover, crop adaptation depends on winter ecotypes (autumn sowing in Mediterranean climates) and spring ecotypes (sowing in colder regions), which condition its agricultural cycle planning [23–26].

The growing food demand underscores the need to develop more sustainable fertilizers, and species such as *Lupinus albus* are presented as ideal candidates for studies in this field, due to their high tolerance to adverse conditions and their ability to improve soil health by fixing nitrogen in symbiosis with bacteria [11, 23, 27]. This study evaluates the impact of fertigation with an organic biofertilizer (ORGAON)^®^ from fruit and vegetable leachate, in combination with PGPB, on the health and productivity of *Lupinus albus* Var. Orden Dorado, using metagenomic techniques to analyze microbial interactions and their effects on agricultural sustainability.

## Methods

### Plant species

Seeds of *Lupinus albus* var. Orden Dorado (Instituto de Investigaciones Agrarias Finca La Orden-Valdesequera, Badajoz, Spain) were used and previously stored in darkness at 4 °C until the beginning of trials. The seeds were superficially sterilized with 70% ethanol (30 s), followed by sterile water-washing. For pregermination, sterilized PVC trays with vermiculite were used, hydrated with sterile water until reaching field capacity (autoclave, 121 °C, 20 min). The seeds, previously hydrated for 12 h at 4 °C, were placed in vermiculite and covered with filter paper. They were kept in darkness at 20 °C ± 2 °C for 96 h until the appearance of a 2 cm ± 0.2 cm radicle.

### PGPB bacterial strains

The PGPB strains were *B. pretiosus* (C1) and *P. agronomica* (C2), described and characterized by Robas Mora et al. [15], belonging to the MICROAMB group’s strain collection. C1, isolated from the rhizosphere of *Medicago sativa*, produces indoleacetic acid (IAA, 5.61 ± 0.03 µg/mL) and siderophores. C2, isolated from free soil, produces IAA (5.85 ± 0.09 µg/mL), 1-aminocyclopropane-1-carboxylate deaminase (ACCd) and siderophores. Both strains were sequenced by Illumina paired-end whole-genome sequencing; reads were quality-filtered and de novo assembled, and the assemblies are deposited as *B. pretiosus* (GenBank: GCA_025916425.1) and *P. agronomica* (GenBank: GCA_025917275.1). Species assignment was confirmed using genome-wide metrics, Average Nucleotide Identity (ANI; FastANI) and digital DNA–DNA hybridization (dDDH; GGDC), adopting standard thresholds (ANI ≥ 95%; dDDH ≥ 70%).

To assess biosafety and genomic traits associated with plant–microbe interactions, the assembled genomes of *B. pretiosus* and *P. agronomica* were analyzed. Several in silico tools were employed, including PGPT-pred and PIFAR-pred in strict mode, using BLASTP and HMMER to predict plant growth-promoting functions and microbial interaction factors. Additionally, the genomes were submitted to the CARD Resistance Gene Identifier (RGI v6.0.1), applying strict criteria and considering only perfect and strict hits with complete genes. Complementary analyses were performed using AMRFinderPlus (v3.12.0) to identify additional antimicrobial resistance genes.

### Irrigation matrices

#### Valorized organic fertilizer (ORGAON^®^PK) and biofertilizer formulation

ORGAON^®^PK, a fertilizer derived from horticultural and fruit waste leachates, with phosphorus (0.10 ± 0.03%) and potassium (2.58 ± 1.52%), at a dilution of 1/512, was used. A UV-sterilized version (ORGAON^®^PK_ST) was included. ORGAON^®^PK was sterilized using a UV-C lamp (254 nm Hg-UV lamp) in sterile conditions inside a laminar flow hood Faster BHEN-2004 (Faster, Cornaredo, MI, Italy) for 1 h in sterile Petri plates (14 cm) filled with 2 mm of ORGAON^®^PK. To check the sterilization, 10 µl from each plate and sampled in standard method agar plates (SMA, Pronadisa^®^, Madrid, Spain) using a Drigalsky spatula. The metagenomic analysis collected in Robas Mora et al. [11] revealed a bacterial community dominated by Firmicutes (38.32%), Proteobacteria (29.09%) and Bacteroidetes (26.73%). Its physicochemical characterization is shown in Supplementary Material 1.

The biofertilizer was prepared by adding 20 mL of a 4 McFarland bacterial suspension of each strain (C1 and C2) per liter of ORGAON^®^PK. The mixture was applied to the vines on a weekly basis and stored at 4 °C until use. For comparison, control fertilizer (CF) and water treatments were also applied to the vines under the same conditions.

#### Chemical fertilizer (CF), irrigation with water (control); application schedule and irrigation regime

To compare the biofertilizer with a conventional synthetic fertilizer, vines were also treated with Universal Fertilizer Complet^®^ (CF; Compo Iberia, Barcelona, Spain). This NPK solution provides 7% nitrogen, which includes nitric, ammonia, and urea forms, as well as 6% phosphorus and 5% potassium. In addition, it contains small amounts of micronutrients such as boron, copper, iron, manganese, molybdenum, and zinc, most of them chelated with EDTA. For control treatments, vines received only sterile tap water, autoclaved at 121 °C and 1 atm for 20 min to maintain osmolarity, with or without the selected PGPBs (C1 and C2).

Once a week, irrigation was carried out up to field capacity (250 mL) with the different irrigation matrices supplemented with the PGPB strains (C1 and C2). In order to maintain the humidity of the soil throughout the three months of growth trial, and to avoid saturation and waterlogging, two or three additional irrigations with water (average experimental volumes of 20 mL) were carried out.

### Experimental design and growth test

The experiment followed a completely randomized design (CRD) with six replicates (pots) per treatment. Each pot (20 cm) contained eight pre-germinated seeds of Lupinus albus var. Golden Order (repeats) on leachate trays of 40 × 35 cm (Table 1), grown under controlled phytotron conditions. Pots were randomized in position and relocated every 48 h to minimize positional effects. The experiment lasted three months under controlled conditions in a phytotron (11 h light, 13 h dark, 18 °C ± 3 °C, 30% ± 5% RH). Each pot was watered independently, and its position in the phytotron was randomized, changing its position every 48 h. After the experiment, the plants were harvested to analyze their biomass. The physicochemical characterization of the soil is shown in Supplementary Material 2.

**Table 1:** Experimental design with six replicates per treatment in *Lupinus albus* var. Gold

TREATMENTS		CHEMICAL			
		Water (control)	Chemical Fertilizer (CF)	ORGAON^®^PK (OPK)	ORGAON^®^PK_ST (OPK_ST)
BIOLOGICAL	Control (without inoculum, C0)	WC0	CFC0	OPKC0	OPKSTC0
	*Bacillus pretiosus* (C1)	WC1	CFC1	OPKC1	OPKSTC1
	*Pseudomonas agronomica* (C2)	WC2	CFC2	OPKC2	OPKSTC2

W, Water; CF, Chemical fertilizer; OPK, ORGAON^®^PK, OPK_ST (ORGAON^®^_ST)

### Extraction of rhizospheric microbial communities

From each pot, 5 g of rhizospheric soil was extracted, which was mixed with a single sample for every 2 pots, obtaining a total of 3 replicates for each treatment with 10 g of soil per replicate. The modified protocol of García-Villaraco et al. (2010) was followed, featuring the suspension of 2 g of soil in saline solution (0.45% NaCl) and a subsequent homogenization at 16,000 r.p.m. with an Omni Mixer Homogenizer for two minutes, followed by a centrifugation at 4000 rpm for 10 min.

### Study of rhizospheric microbial communities

#### Community antibiotic resistance profile (cenoantibiogram)

Community antibiotic resistance was evaluated in triplicate by inoculating the bacterial extract on Mueller-Hinton agar (Condalab^®^, Madrid, Spain) and using Etest^®^ strips (BioMérieux^®^, Marcy l’Etoile, France). The antibiotics analyzed were amoxicillin (AML), amoxicillin/clavulanic (AUG), cefotaxime (CTX), piperacillin (PP), cefepime (PM), piperacillin/tazobactam (TZP), imipenem (IMI), trimethoprim/sulfamethoxazole (TS), imipenem/imipenem + EDTA (IMD), gentamycin (CN), nalidixic acid (NA), and ciprofloxacin (CIP). It was analyzed by PCA using SPSS v.29.0.

#### Metabolic diversity of the microbial community: Shannon-Weaver index

Functional diversity of the rhizosphere communities was determined with Biolog EcoPlates^®^ (Biolog Inc., Hayward, CA, USA), each containing 31 carbon sources in triplicate plus a negative control. The bacterial extract described above was adjusted to 0.5 McFarland with sterile saline (0.45% NaCl); 150 µL of this suspension were dispensed into each well, and plates were incubated at 25 °C for seven days. Optical density (OD₅₉₅) was recorded every 24 h with a Multiskan FC microplate reader (Thermo Fisher Scientific). For each reading the corresponding blank value was subtracted, and triplicate means were calculated for each substrate. Average Well Colour Development (AWCD) was plotted against incubation time to obtain metabolic-activity curves for each community, and the time point of maximum AWCD was used to compute functional-diversity indices.

Metabolic diversity was expressed as the Shannon–Weaver index (Hₘ): Hm = –∑ qi log₂(qi); where qi is the proportion of corrected absorbance for well i: qi = Ai / ∑Ai; with Ai being the blank-corrected absorbance of well i and ∑Ai the summed absorbance for the plate at that time point. The index was calculated for each biological replicate, and the resulting values were subjected to statistical analysis to detect significant differences among treatments.

#### Taxonomic diversity of the microbial community: alpha and beta diversity

Microbial DNA was extracted from rhizospheric soil samples using the QIAsymphony PowerFecal ProDNA Kit (QIAGEN N.V., Venlo, Netherlands). The hypervariable V3-V4 region of the 16 S rRNA gene was amplified using the primer pair 341 F (5′-CCTACGGGNGGCWGCAG-3′) and 805R (5′-GACTACHVGGGTATCTAATCC-3′) (Klindworth et al., 2013), following the Illumina protocol 15,044,223 B for 16 S rRNA gene metagenomic library preparation. The initial PCR incorporated a universal linker sequence to facilitate the addition of Nextera XT indices (Illumina Inc., San Diego, CA, USA) in a subsequent indexing PCR.

Amplicon libraries were quantified using the Quant-iT™ PicoGreen™ dsDNA assay kit (Thermo Fisher Scientific, Waltham, MA, USA), pooled equimolarly, and the quality and quantity were validated using the Bioanalyzer 2100 (Agilent Technologies, Santa Clara, CA, USA) and the Library Quantification Kit for Illumina (Kapa Biosystems, Wilmington, MA, USA), respectively. The pooled libraries were sequenced on an Illumina MiSeq platform using a paired-end 2 × 250 cycle configuration with MiSeq Control Software v2.6.2.1, including 15% PhiX control to improve sequence diversity.

Raw reads were processed using QIIME2 v2021.11. DADA2 was employed for quality filtering, denoising, and chimera removal. Taxonomic assignment was carried out using a naïve Bayes classifier trained on the SILVA 138_99 reference database, trimmed to the V3-V4 region.

Alpha diversity was evaluated using the Observed Features, Shannon, Faith’s Phylogenetic Diversity, and Pielou’s Evenness indices. Beta diversity was assessed using weighted UniFrac distance matrices. Kruskal-Wallis tests were used to evaluate statistical differences in alpha diversity, while PERMANOVA was applied to beta diversity comparisons.

Alpha diversity summarizes within-sample diversity. Used indices include: (i) Observed Features (ASV richness) to capture the number of detected taxa; (ii) Shannon to weight both richness and evenness; (iii) Pielou’s evenness to isolate community evenness; and (iv) Faith’s Phylogenetic Diversity to account for the evolutionary breadth of lineages present. These complementary indices are well suited to rhizosphere datasets where treatments may alter both taxon counts and their relative balance, and where phylogenetic breadth can change even when taxonomic counts are similar. For between-sample structure weighted UniFrac was used, a phylogeny-aware distance that emphasizes abundance-weighted differences along the 16 S rRNA gene tree. Its employment is appropriate here because OPK and PGPB treatments are expected to shift relative abundances within core rhizosphere clades rather than cause wholesale taxon turnover. Prior to diversity analyses, the feature table was normalized to mitigate library-size effects.

Group differences in α-diversity were tested with Kruskal Wallis (non-parametric and robust to non-normality typical of ecological indices), while community-level differences in β-diversity were tested with PERMANOVA on the UniFrac matrix. Beta diversity was tested with PERMANOVA (QIIME2 v2021.11; q2-diversity beta-group-significance, method = permanova, 999 permutations) on weighted UniFrac distance matrices to evaluate whether community centroids differ among irrigation matrices (water, CF, OPK, OPK_ST), inoculation (C0, C1, C2), and their interaction. This approach is appropriate for 16 S rRNA gene V3–V4 amplicon data because weighted UniFrac incorporates phylogeny and relative abundance, capturing abundance-weighted compositional shifts beyond alpha diversity. The homogeneity of dispersions was verified with PERMDISP (999 permutations). When overall PERMANOVA was significant, a pairwise PERMANOVA was ran with Benjamini–Hochberg FDR correction and pseudo-F, R² (effect size) and adjusted P-values were reported. The aim was to determine whether fertilizer matrix and/or PGPB inoculation drive statistically significant shifts in rhizosphere community composition. Together, these analyses indicate whether OPK ± PGPB alters within-sample diversity (richness/evenness/phylogenetic breadth) and between-sample community structure, linking microbiome shifts to the observed functional outcomes (biomass gains and reduced community-level MICs) relative to CF and water controls.

### Plant quality analysis

#### Biometrics

Root and shoot length (cm) as well as the total root and shoot weight (g) were evaluated. Measurements were taken after harvesting and rinsing with distilled water, and dry weight was determined after nine days of drying (20 ± 2 °C).

#### Nutritional quality

Within 24 h of harvest, samples (2 g of wet biomass) were sent to Rock River Labs Spain (Lalín, Pontevedra) for nutritional analysis by near-infrared spectroscopy (NIRS; DS2500, FOSS Analytical, Denmark). Plant material was dried, separated into leaf and stem fractions, and ground to 1 mm with a Cyclotec mill (FOSS). Spectra were collected from 400 to 2 498 nm and processed using Rock River Laboratory proprietary calibrations, which link absorbance patterns at specific wavelengths with reference wet-chemistry values. This allowed the quantification of nutritional traits including crude protein, soluble protein, total amino acids, water-soluble carbohydrates (WSC), fiber fractions (aNDFₘₒ, uNDF₂₄₀), and fiber digestibility (TTFDND). Each sample was analyzed in triplicate to ensure reproducibility.

### Statistical analysis

Analysis of variance (ANOVA) was applied to identify significant differences between irrigation treatments (water, CF, raw/sterilized ORGAON^®^) and the presence of PGPB (C1, C2), considering a significant threshold of *p* < 0.05. Post-hoc comparisons were made using the Duncan test. Additionally, a principal component analysis (PCA) was applied as an unsupervised ordination to summarize multivariate patterns and visualize treatment clustering across several data blocks. Sample scores (PC1–PC2) were plotted with 95% confidence ellipses by treatment to assess grouping. Analyses were performed in SPSS^®^ Statistics AmosTM v.27.0 software.

## Results

### Genomic insights into plant–microbe interactions in two PGPB strains

*B. pretiosus* was analyzed using the PGPT-pred and PIFAR-pred tools under strict mode (BLASTP + HMMER), resulting in the identification of 2133 functions classified as indirect effects, associated with categories such as biocontrol, nutrient competition, secretion of bioactive compounds, and stimulation of the plant immune response, along with 893 functions related to direct mechanisms, including auxin (IAA) biosynthesis, siderophore production, phosphate acquisition, and nitrogen fixation, with major representation in the categories of colonization of the plant system (25%), competitive exclusion (24%), stress-related biocontrol (20%), biofertilization (11%), and phytohormone production (9%).

Specific functional pathways related to phytohormone synthesis and metabolism were also identified, including genes involved in the biosynthesis and transport of indole-3-acetic acid (IAA) via TRP-dependent pathways, genes associated with the degradation of abscisic acid (ABA), functions linked to the biosynthesis and metabolism of gamma-aminobutyric acid (GABA), pathways related to jasmonate production including jasmonic and isojasmonic acids, and additional functions implicated in the stimulation of plant branching and germination processes.

In the PIFAR-pred analysis, plant–microbe interaction factors were distributed across the following categories: toxins (29%), extracellular polysaccharides EPS (24%), detoxification (12%), phytohormone production (11%), multidrug resistance MDRs (5%), and metabolism (5%), as well as specific functions including adhesion (11 genes), plant cell wall degrading enzymes PCWDE (9 genes), motility, proteases, pigments, volatile compounds, and surface structures such as LPS.

The phytohormone production category (11%) was represented by genes involved in the biosynthesis of indole-3-acetic acid (IAA) via TRP-dependent pathways, cytokinins (*ipt*, *ptz*), salicylic acid (*salicylic hydroxylase*), and compounds related to jasmonate signaling, suggesting a potential to modulate plant development and physiological responses.

*Pseudomonas agronomica* was analyzed using PGPT-pred in strict mode (BLASTP + HMMER), identifying functions distributed across several functional categories, with the most prominent being colonization of the plant system (26%), competitive exclusion (21%), stress control and biocontrol (20%), biofertilization (13%), phytohormone production and plant signaling (10%), bioremediation (8%), and stimulation of the plant immune response (1%), while putative functions with no defined classification accounted for the remaining 1%.

Additionally, specific functional pathways related to hormone synthesis and metabolism were identified, including 82 genes involved in the biosynthesis and transport of indole-3-acetic acid (IAA) through multiple TRP-dependent routes, 48 genes associated with gamma-aminobutyric acid (GABA) metabolism, 34 genes implicated in cytokinin production and transport, 20 genes related to xanthine metabolism, and 2 genes linked to jasmonate biosynthesis. No functions directly associated with abscisic acid (ABA) degradation were detected in this analysis.

In the PIFAR-pred analysis, plant–microbe interaction factors were distributed across the following categories: toxins (44%), extracellular polysaccharides EPS (14%), detoxification mechanisms (10%), phytohormone production and signaling (9%), adhesion (5%), multidrug resistance MDRs (5%), metabolism (3%), and motility (3%), along with individual functions related to pigment biosynthesis, LPS production, proteases, volatile compounds, and plant cell wall degrading enzymes PCWDE, each representing 1% or less of the annotated genes.

## Genomic assessment of antibiotic resistance determinants

The genomic analysis of *B. pretiosus* and *P. agronomica* using CARD and AMRFinderPlus revealed no perfect hits and no evidence of mobile genetic elements encoding transmissible antibiotic resistance determinants, including β-lactamases or aminoglycoside-modifying enzymes. Only chromosomally encoded resistance-associated genes were detected, including efflux pumps and target protection mechanisms, classified as “strict hits” by CARD. These findings were consistent across both tools and support the absence of horizontally transferable resistance genes in either strain.

## Analysis of metabolic activity

The taxonomic diversity and metabolic activity of the soil microbiota are key indicators to assess the potential of soil in the transformation of organic matter. These factors indirectly influence the roots’ ability to absorb nutrients, as greater functional diversity indicates a more robust and efficient microbial ecosystem. To measure this diversity, the Shannon Index was used, which quantifies both the richness and evenness of the species present in the microbial community.

**Fig. 1:**
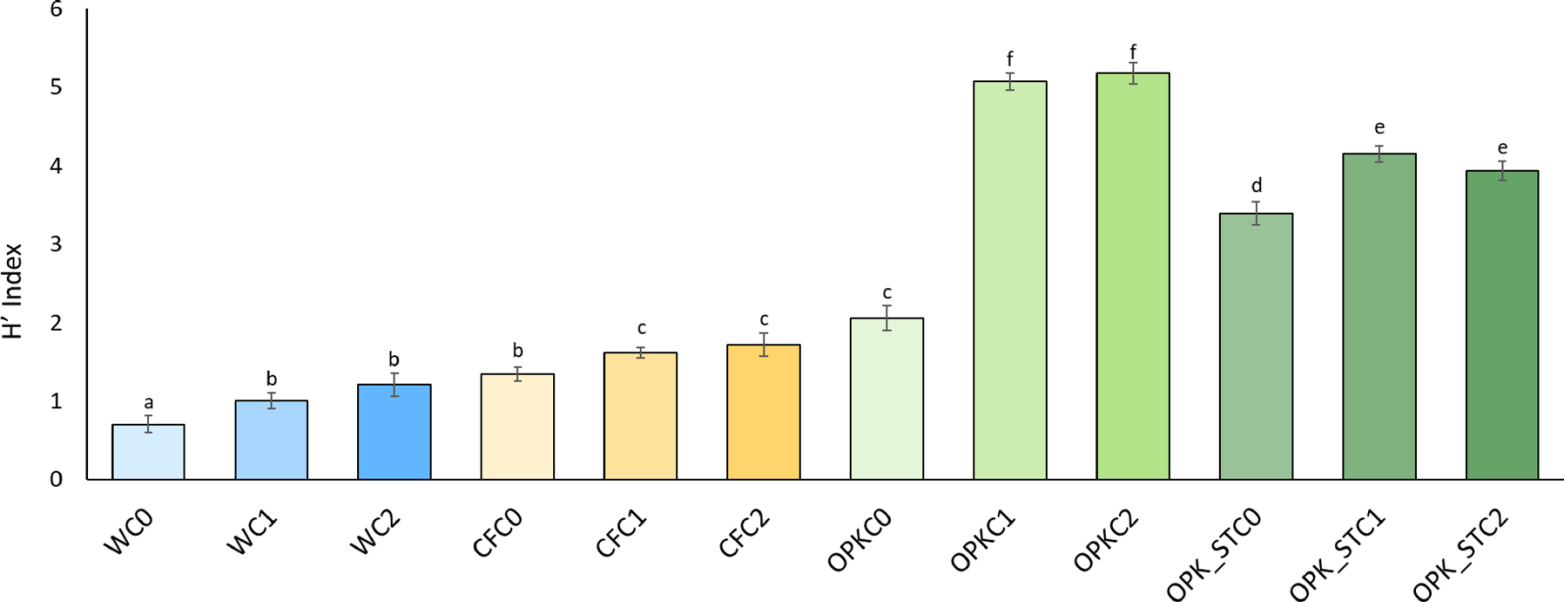
shows that, after an incubation of 120 h, the addition of the strains *B. pretiosus* (C1) and *P. agronomica* (C2) induces a significant increase in the values of the Shannon Index in comparison to the controls. These higher values are recorded regardless of the chemical matrix (biofertilizer or chemical fertilizer) on which the strains are added, suggesting that their positive effect on microbial diversity is common to all test conditions

Figure 1. Shannon Index (H’) of metabolic diversity by Biolog EcoPlate™ of the rhizospheric community after 120 h of treatment. ANOVA (Duncan *post hoc*) of the average values per treatment (*n* = 3) are shown. Bars with identical letters do not show significant differences between the mean values (*p* < 0.05). The treatments are coded as follows: C0, inoculum-free control; C1, *B. pretiosus*; C2, *P. agronomica*; W, irrigation with water; CF, irrigation with chemical fertilizer; OPK, irrigation with ORGAON^®^PK.

## Antibiotic resistance analysis

The cenoantibiogram analysis indicated that the introduction of PGPBs, independently of the chemical carrier used, consistently reduced MIC values compared with the corresponding controls (ANOVA). Detailed results of the statistical analyses, together with the MIC values of the soil bacterial community under the different fertigation treatments, are provided in Supplementary Material 3. Notably, a significant decrease in MIC values (*p* < 0.01) was observed in most treatments involving inoculation with strains C1 or C2, relative to uninoculated controls (C0).

**Fig. 2:**
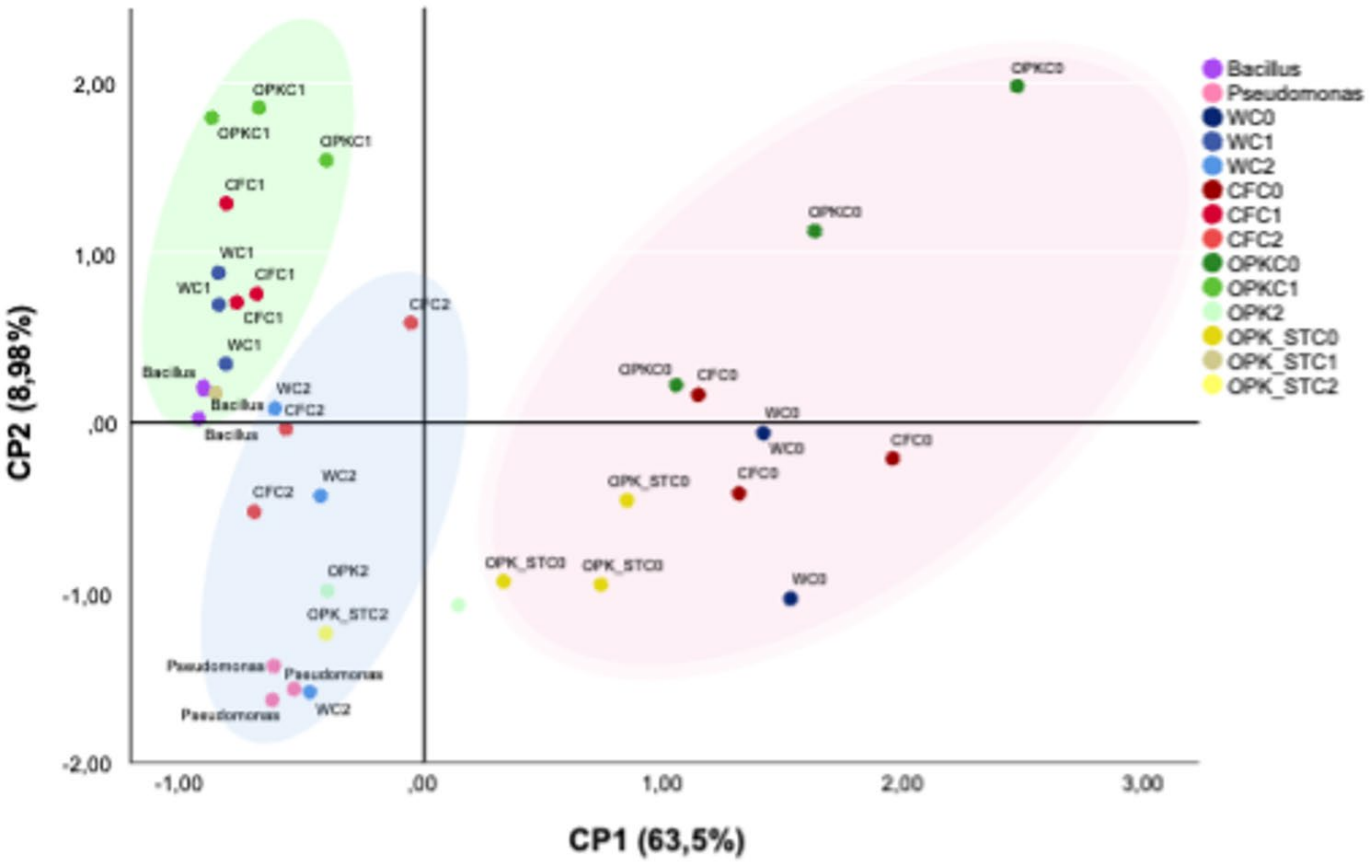
Análisis de componentes principales (ACP) que muestra la distribución y variación de los tratamientos de riego (químico y biológico) en función de los dos componentes principales que explican el modelo. CP1 representa el 63,5% de la varianza, mientras que CP2 representa el 8,98%. Las variables analizadas corresponden a la CMI (Concentración Mínima Inhibitoria) del cenoantibiograma de cada suelo. Los tratamientos se codifican como: C0 (control, sin inóculo), C1 (*B. pretiosus*), C2 (*P. agronomica*), W (riego), CF (fertilizante químico) y OPK (ORGAON)^®^PK). En la Fig. 1, los controles sin inóculo están en gris, mientras que las muestras tratadas con C1 se agrupan bajo la elipse púrpura y las muestras tratadas con C2 bajo la elipse rosa

To explore the clusters or trends of variation of the treated soils according to the MIC values of the rhizospheric communities obtained in the cenoantibiograms, an ACP and its representation in the two-dimensional plane were carried out. The three main variables that best explained the model were CP1: CN-gentamicin (63.50%); CP2: Amoxicillin-AML (8.98%); and CP3: Piperacillin-PP (8.51%), which together account for 80.84% of the model variance. For a better understanding, Fig. 2 shows the spatial distribution of the two main components (CP1 and CP2).

When jointly analyzing the response of rhizospheric communities, affected by the different chemical-biological treatments, to the antibiotics most widely used in human clinics, it is observed that the factor that exerts the greatest influence on the pooling of data is the addition of PGPB. Thus, soils with chemical treatments without inoculum are predominantly grouped in the right half of the graph, while those supplemented are mostly to the left of the abscissa axis. Likewise, a grouping of the antibiotic resistance profiles of the rhizospheric communities of plants treated with C2 towards the upper left quadrant is evidenced in all chemical treatments. Similarly, the antibiotic resistance profiles of plants treated with C1 tend to cluster in the lower left quadrant. In both cases, the influence that the PGPB strain used exerts on the rhizospheric community that hosts it is evidenced, reducing MIC compared to antibiotics used most in human clinics.

## Structure and dynamics of the microbial community in response to treatments

In the metagenomic analysis, a total of 648,953 valid reads were obtained from the sequencing of the 16 S rRNA gene amplicon after clipping (SAMN 40643627; PRJNA 1092974). The mean amplicon length was 301 bp. The number of valid sequences detected for each soil sample exceeded 14,171. The alpha rarefaction curve was flat, indicating that the genetic data were sufficient for a reasonable estimate of the total taxonomic composition. Analyzing the quantitative and qualitative composition of the different rhizospheric communities, no notable differences or a displacement of the taxa of an edaphic microbial community were observed compared to the rest of the analyzed samples.

The PERMANOVA analysis did not show a significant difference in the structure of the soil microbial community in relation to the different types of fertilizers used (*p* = 0.581) nor in relation to the different strains used. Similarly, analysis of the alpha diversity index of the microbial community in the soil in response to each treatment did not show that OTU diversity and richness differed significantly from the respective controls. Moreover, the results of the community structure analysis (ADONIS) indicate that neither the application of fertilizers nor the presence of bacteria have a significant effect on the structure of the soil microbial community (Fertilizer: F (2, 7) = 0.861, *p* = 0.615; Bacteria: F (2, 7) = 0.813, *p* = 0.687). Most of the variability in soil microbial community structure is represented by variability within samples (R2 = 0.676), which is shown in Supplementary Material 4.

In the ACP graph of the beta diversity analysis, using the weighted Unifrac metric, it was shown that different treatments had different effects on the taxonomic structure of microorganisms in the soil. In the same sense, the analyses carried out using ANCOM-BC revealed statistically significant differences in the microbial composition between the different treatment groups. Specifically, it was observed that most of these variations were associated with unculturable bacteria, with a minority of identifiable genera. It is noteworthy that these identified genera did not represent a significant proportion within the microbial community studied. Specific diversity metrics and graphs are detailed in Supplementary Material 5.

**Fig. 3:**
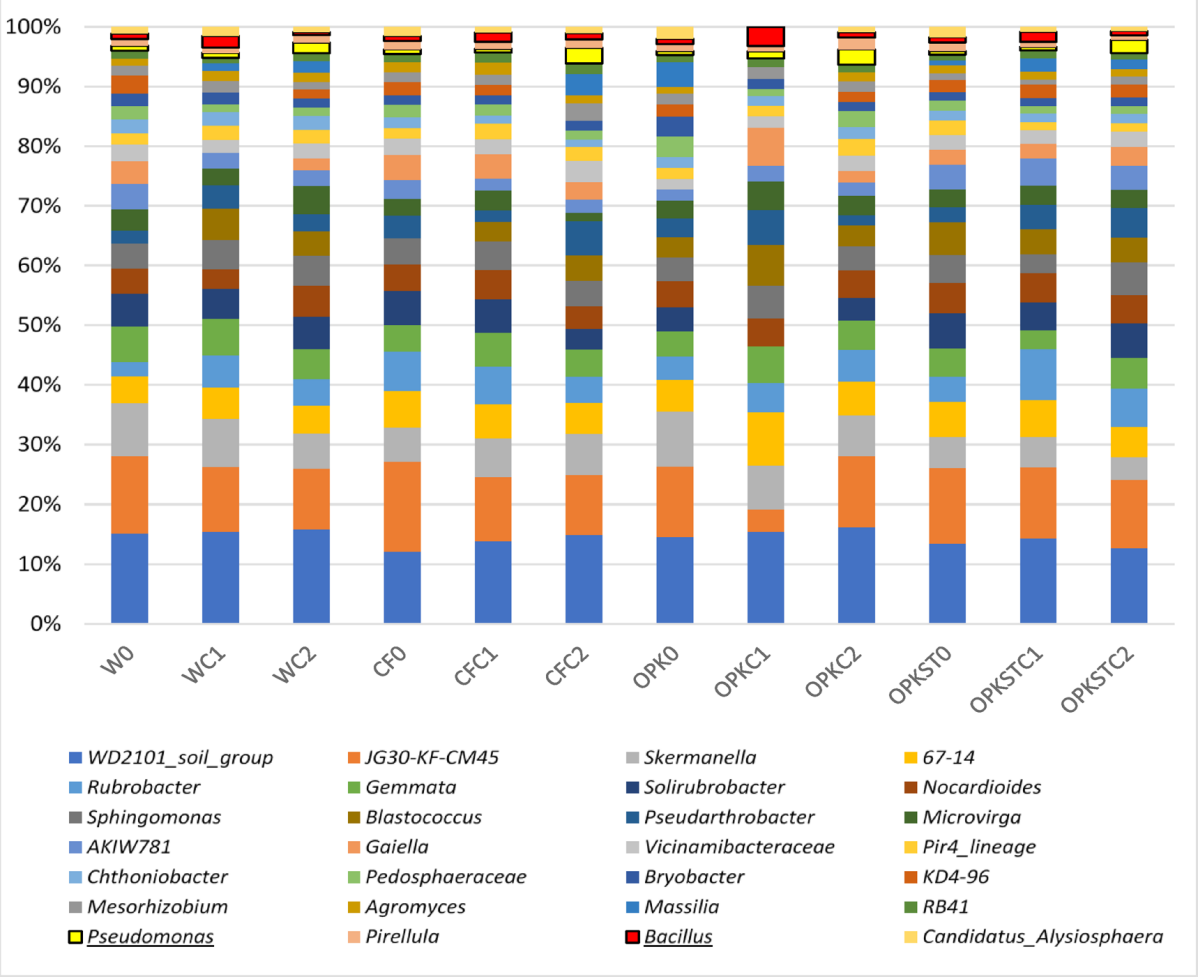
Variation in microbial composition between treatments: analysis of absolute abundances of OTUs

In relation to the distribution and proportion of taxa, the metagenomics results show a greater portion of the taxon *Bacillus* in those soils treated with C1, just as the taxon *Pseudomonas* is more represented in soils treated with C2. This fact is shown in soils subjected to any of the chemical fertilization treatments used to transport bacteria. Such relative increases in *Bacillus* and *Pseudomonas* taxa, as appropriate, could evidence the survival of the added strains (Fig. 3).

## Nutritional and biometric parameters

**Fig. 4:**
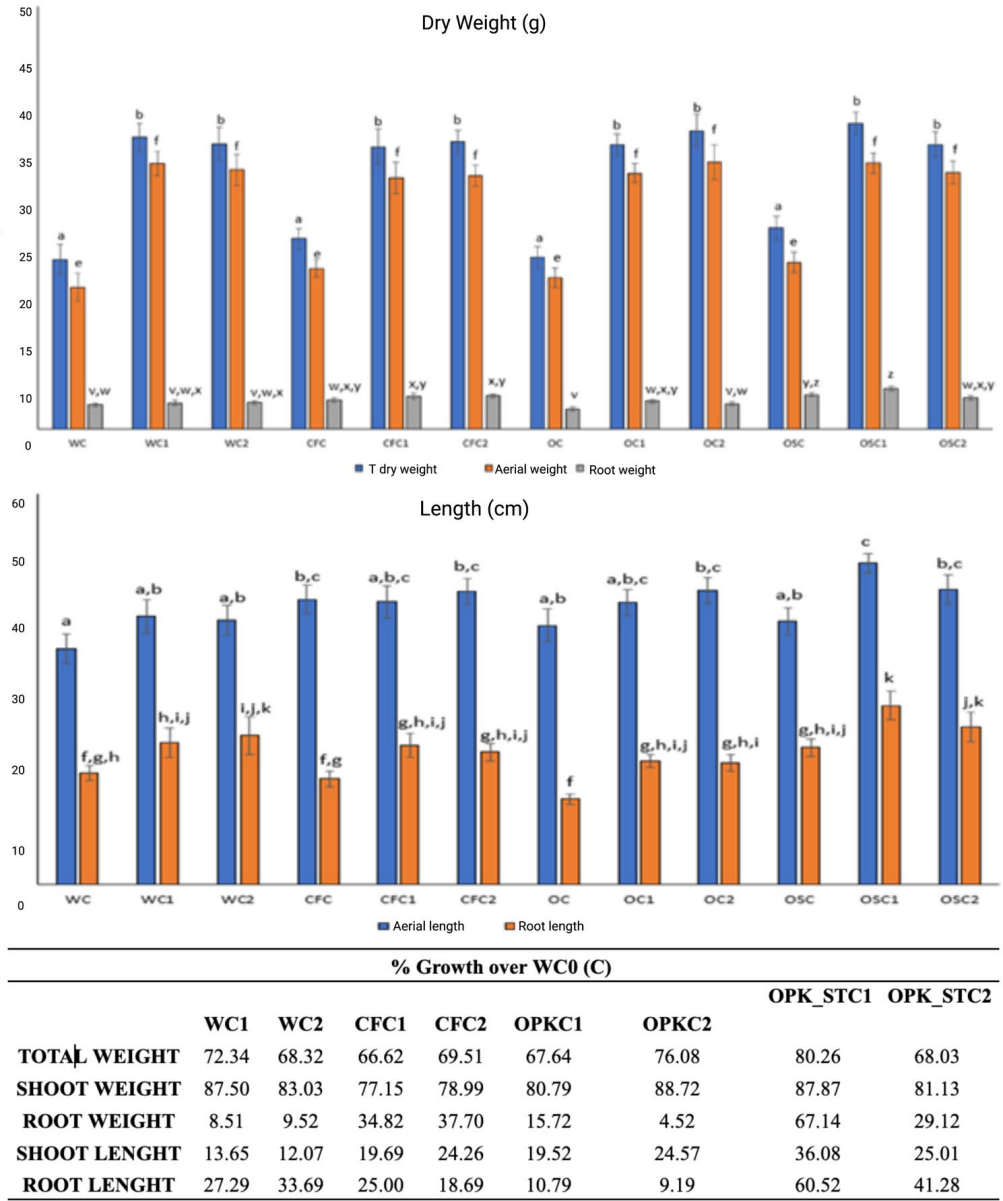
**A** Datos de análisis biométrico relacionados con el peso seco (g) de Lupinus albus. Valores medios para *n* = 3. Las barras con letras idénticas indican que los valores medios no son significativamente diferentes (*valor p* < 0,05). Codificación de letras: [ac] representa las comparaciones de medias para el tratamiento, **B** datos de análisis biométrico relacionados con el peso seco (g) de Lupinus albus. Valores medios para *n* = 3. Las barras con letras idénticas indican que los valores medios no son significativamente diferentes (valor p < 0,05). Codificación de letras: [ac] representa las comparaciones de medias para el tratamiento, **C** porcentaje de aumento en los parámetros biométricos de Lupinus albus en relación con el control de agua sin inocular (WC0)

After assessing the effects of PGPB and chemical treatments on soil microbial communities, their impact on the host plants was evaluated. Biometric variables associated with plant dry weight, including both individual fractions and total biomass, exhibited a consistent pattern. No significant differences were detected among the chemical treatments alone. In contrast, inoculation with bacterial strains significantly enhanced shoot dry weight, total dry weight, shoot length, and shoot elongation. Root dry weight, however, remained unaffected by bacterial inoculation (Fig. 4).

Treatments without microbial inoculation (W, CF, and ORGAON^®^PK-st) exhibited lower crude protein content. In contrast, inoculation with *Pseudomonas agronomica* (C2) in ORGAON^®^PK and ORGAON^®^PK-st significantly increased total crude protein and starch levels. *B. pretiosus* (C1) enhanced methionine, FDA, and aFDA concentrations across all treatments. Both strains (C1 and C2) consistently promoted higher tFDND, sugar, and fatty acid content in all irrigation matrices. Furthermore, *B. pretiosus* (C1) reduced lignin content in ORGAON^®^PK and ORGAON^®^PK-st. No significant differences were observed in mineral uptake between biological treatments. Detailed biometric data as shown in Supplementary Material 6.

**Fig. 5:**
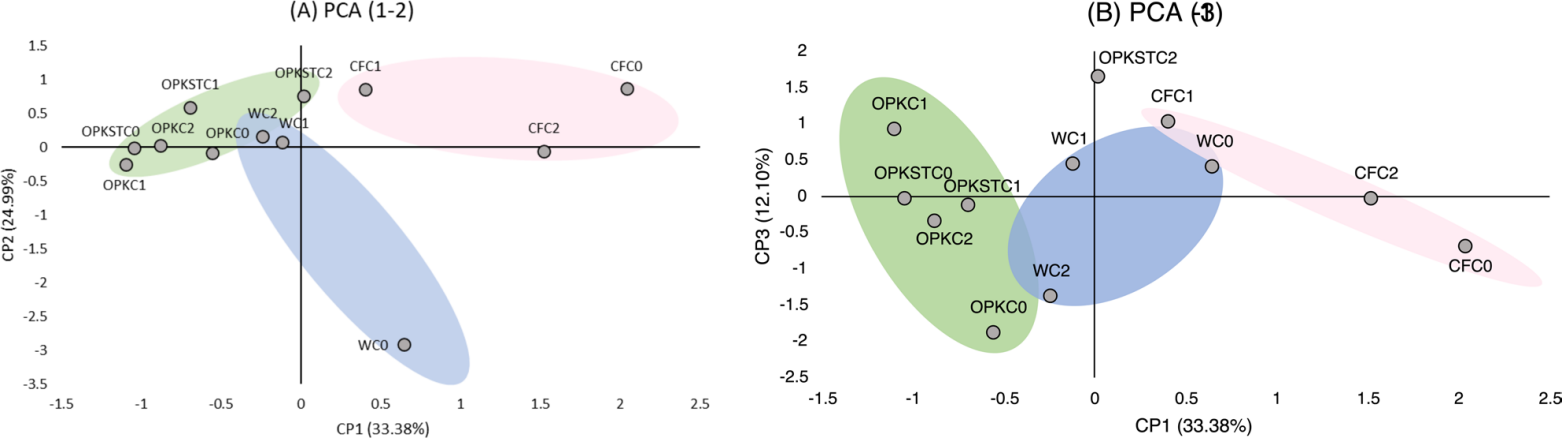
Análisis de Componentes Principales (ACP) de variables nutricionales. **a** CP1 (Proteína Cruda, que explica el 33,38% de la varianza) y CP2 (aminoácidos totales, que explica el 24,99% de la varianza), **b** CP1 (Proteína Cruda, que explica el 24,99% de la varianza) y CP3 (Proteína Soluble, que explica el 12,10% de la varianza)

The PCA used to reduce the dimensionality of the data (Fig. 5) reveals that the explanation of the model requires the inclusion of at least the first three components (CP1: 33.38%, CP2: 24.99%, CP3: 12.10%), which accumulate a variance of 70.47%.

The distribution of soils considering CP1 and CP3 (Fig. 5a) shows aggregation according to the chemical matrix of the fertilizer. The same results are obtained when the graph considers CP1 and CP3 (Fig. 5b). Therefore, the PCA shows that chemical treatment exerts a greater influence on the model, which explains the plant’s behavior. PGPBs contribute to the chemical matrix to promote its transformation and absorption by the plant and stimulate both directly and indirectly (phytohormone production) plant growth.

## Discussion

The functional characterization of *B. pretiosus* and *P. agronomica* using PGPT-pred and PIFAR-pred provides mechanistic evidence supporting their observed effects as plant growth-promoting bacteria (PGPB) and their ecological behavior in the soil. Both strains showed highly represented functional categories related to competitive exclusion, secretion of bioactive compounds, and stress control, which are consistent with their ability to persist in the rhizosphere and modulate microbial communities. Similar to previous findings on PGPB-mediated microbial reshaping in legumes [28], our results confirm that functional versatility underlies ecological persistence. In contrast, few studies have explicitly linked these traits with reduced antibiotic resistance levels in the rhizosphere, making our observations novel in this regard. These traits may help explain the observed reduction in antibiotic resistance levels within the rhizospheric microbiota, particularly considering the abundance of effector systems and detoxification mechanisms involved in microbial competition and defense. Previous studies have shown that genes related to hormone metabolism are enriched in leaf-associated plant growth-promoting bacteria, while carbohydrate metabolism and antibiotic genes are enriched in soil-associated bacteria [19].

Hormonal biosynthesis and signaling pathways were particularly enriched in *P. agronomica*, including multiple TRP-dependent auxin pathways, as well as genes involved in the metabolism of cytokinins, GABA, ABA, and jasmonates. This suggests a multi-hormonal regulatory potential that could explain the enhanced root development and aerial biomass observed in vivo, in line with previous findings [29, 30]. *B. pretiosus*, on the other hand, displayed a more balanced functional profile, supporting both direct mechanisms (e.g., nitrogen fixation, phosphate solubilization) and indirect ones (e.g., biofilm formation, antimicrobial secretion), a versatility commonly associated with species adapted to complex soil environments [31]. Moreover, neither strain showed genomic signatures associated with virulence or transferable antibiotic resistance, reinforcing their safety and suitability as bioinoculants [32, 33]. This agrees with studies of other environmentally safe *Bacillus* inoculants [29], but differs from those reporting mobile ARGs in field-applied Pseudomonas [34, 35]. The combination of PGPT and PIFAR tools enabled linking plant- and community-level phenotypic observations to their genomic basis. These findings consolidate the role of *B. pretiosus* and *P. agronomica* as next-generation biofertilizers with strong potential for sustainable agriculture.

The introduction of a foreign organism can reduce the functional or metabolic diversity of a system [36]. However, this is not a universal rule. The most frequently assessed aspects of diversity are species richness (or number), and the proportional distribution of the number of individuals of each species. These measurements are a way of describing ecological communities, in terms of dominance or equity, as another component of diversity. In the field of microbial ecology, many indices for measuring biodiversity have been proposed and are widely available [37, 38]. Thus, the introduction of a new taxon can even induce an increase in this diversity as long as there is no displacement of the rest of the taxa in the community [39]. This fact seems to be occurring in all soils treated both with *B. pretiosus* and *P. agronomica* regardless of the irrigation matrix used.

Genetic diversity follows the same pattern and represents a critical factor in understanding ecosystem dynamics, particularly biogeochemical cycling [40]. Nevertheless, this component was overlooked for years in the scientific literature, due to the lack of technical tools that would allow analyzing and modeling the role of microorganisms in ecosystems [41]. Recent advances, such as amplicon metagenomics, have paved the way for the unprecedented growth of ecological studies of microorganisms in their natural environments [42, 43]. In the present paper, we found a remarkable abundance of species belonging to several genera of bacteria, such as *Balneimonas*, *Kaistobacter*, *Nocardiodes*, *Arthrobacter*, *Streptomyces*, *Mycobacterium* and others of ecological and agricultural importance. These microorganisms generally display slow growth rates and may be recalcitrant or even uncultivable in conventional culture systems. Accordingly, its presence in the soil implies richness and great diversity, but to reveal its phenotypic response wider studies in the field of proteomics and metabolomics are needed. On the contrary, the species of the genera *Bacillus* and *Pseudomonas* do behave as fast growers, which is why their presence in soil does have a very notable effect on the phenotype of the soil microbiota; both in the profile of antibiotic resistance and functional diversity. This behavior is even more noticeable in soils that have been irrigated by *Pseudomonas agronomica*. Along these lines, our study revealed significant differences in microbial composition between the different treatment groups, according to the analysis carried out using ANCOM-BC. Surprisingly, most of these differences were associated with unculturable bacteria, suggesting the presence of a rich microbial diversity not yet characterized in the studied soil. The findings underscore that a deeper understanding of soil microbial diversity could inform microbial ecology and unlock practical applications in agriculture, bioremediation, and sustainability [11, 25, 44, 45]. Given the growing demand for environmentally friendly farming practices, it is crucial to ensure that products used to improve soil fertility do not disturb natural microbial diversity. The observation that significant differences in microbial composition between the treatment groups were mainly associated with unculturable bacteria suggests that the biofertilizers used in this study did not exert any negative impacts, nor reduce diversity, or alter the overall structure of the rhizospheric microbial community. This discovery reinforces their potential as effective tools to improve soil fertility. Similarly, the application of different alpha and beta diversity indices revealed that the use of biofertilizers did not affect the richness and distribution of microbial species in soils treated with PGPB, confirming their suitability to promote environmental sustainability, especially in agricultural soils [9, 46].

Analysis of the relative abundance of taxa in rhizospheric soils revealed a higher prevalence of *Bacillus* in soils treated with C1, and an increased representation of *Pseudomonas* in soils treated with C2. This result suggests that PGPBs administered in their biofertilizers (*B. pretiosus* and *P. agronomic*) seem to have persisted in the rhizosphere of *Lupinus albus* Var. Orden Dorado. The ability of these strains to colonize the soil and maintain itself over time is an encouraging result, as it suggests their potential usefulness in improving soil fertility and in other agricultural applications. However, to gain a competitive advantage in soil, some microorganisms are able to produce antimicrobial compounds to inhibit the growth of their competitors [47, 48]. Furthermore, antimicrobial-producing microorganisms may possess self-protection or antimicrobial resistance mechanisms, which allow them to effectively defend themselves against the action of antimicrobial compounds [49]. Thus, the addition of this type of bacteria can induce changes in the behavior of the soil microbiota that hosts them. The trial showed a significant reduction in these MICs of the welcoming rhizospheric communities of the C1 and C2 strains compared to all analyzed antibiotics commonly used in human clinics. In this way, all soils to which a bacterium has been added, through fertigation treatment, have been displaced in the projection of the ACP versus the media without the addition of bacteria. This fact, still understudied in communities, has already been described by our group [20]. In this same sense, the principal component analysis shows that the variables with the greatest statistical weight are beta-lactam antibiotics (amoxicillin and piperacillin), as well as the aminoglycoside and gentamicin. Resistance mechanisms in this group often involve the production of transmissible inactivating enzymes. This suggests a bioprotective role for *B. pretiosus* and *P. agronomica* and indicates that inoculation with these strains may also limit the transfer of resistance traits to other rhizospheric bacteria. Despite the reduction in MIC, we must continue to understand that the soil will always behave as a reservoir of antibiotic resistance mechanisms. Cooperative interactions between bacteria include processes such as metabolite exchanges, while the production of compounds with antimicrobial activity corresponds to competitive interactions [50]. For this reason, the exogenous addition of PGPBs that do not carry transmissible resistance mechanisms can help to minimize the growing problem of selection and dispersion of resistance mechanisms from their origin in agricultural food production, through the food chain and to the human species [51].

Genome sequence analysis confirmed the absence of mobile genetic elements encoding antibiotic-inactivating enzymes in both *B. pretiosus* and *P. agronomica*. While both strains port some chromosomally encoded resistance-associated genes—mainly efflux pumps and target protection mechanisms—no evidence of transmissible resistance determinants or β-lactamase genes was found. Taken together, the genomic evidence implies that the lower MICs observed in rhizospheric communities arise from ecological interactions, such as competition and niche partitioning, rather than from dissemination of resistance determinants. This ecological interpretation is reinforced by the ability of both strains to modulate the composition and function of native soil microbiota, potentially limiting the spread of resistance determinants. Competitive interactions, such as the production of antimicrobial compounds, and cooperative mechanisms like metabolite exchange, may explain how these strains reduce the risk of horizontal gene transfer within the rhizosphere [19, 52]. In this context, the exogenous addition of PGPBs that lack transmissible resistance genes, such as those characterized here, may serve as a bioprotective strategy to mitigate the environmental propagation of antimicrobial resistance from agricultural systems to the food chain and, ultimately, to human populations [53].

In regard of the effects of chemical/biological treatments on plants, the obtained results suggest that *B. pretiosus* and *P. agronomica* promote biomass accumulation in *L. albus* var. Golden Order, independent of the fertilization matrix, similar to the work of Raglin et al. [54], who also observed enhanced biomass under PGPB inoculation. Interestingly, in our case biomass increases were accompanied by a reduction in rhizospheric MICs, a dual effect not previously described. This dual benefit constitutes one of the most crucial insights of our study. This improvement is due to two factors: firstly, the expression of its PGP activities and, secondly, the fragmentation of organic compounds into simpler elements, which allows the availability of ORGAON^®^PK nutrients to plant roots. Root growth is of great importance as it regulates the metabolic costs of soil exploration, axial and radial transport of soil resources, interactions with soil organisms, and soil penetration [55]. Consequently, the plant can increase the uptake of water and nutrients, favoring the retention of nutrients that could be lost through leaching, and consequently improving its yield [56]. This may be due to the production of auxins, specifically IAA [57]. The obtained results align with those shown by [11], which describe *B. pretiosus* and *P. agronomica* as bacteria that stimulate plant growth by increasing the length and number of roots [15]; especially in chemical treatments with ORGAON^®^PK-st added with both PGPBs.

In relation to nutritional quality, lupins are characterized by high levels of crude protein (between 33 and 47%) and metabolizable energy, which make them highly attractive to integrate concentrated rations for cattle [58]. In the performed experiment, the concentration of crude protein was higher for treatment with ORGAON^®^PK. Protein production performance is optimal when *Pseudomonas agronomica* is used in addition to chemical treatment, a result matching those obtained in other agricultural production plants [59]. Furthermore, methionine is an essential amino acid that has important functions in the growth and development of plants. It allows the synthesis of proteins, helps to eliminate heavy metals and other toxic compounds from soil and plant cells and improves nutritional quality. Animals cannot synthesize methionine and must obtain it from the diet [60]. *Lupinus albus* is a plant species with a low methionine content [61]. No significant differences in methionine concentration were observed among plants treated with different chemical fertilizers. Nevertheless, significant differences were obtained for all treatments supplemented with *B. pretiosus*; especially for the ORGAON^®^PK-st matrix treatment, in line with other authors. Previous studies in other crops suggest that consortia of *Bacillus* strains may enhance methionine content by as much as 50% [62]. Therefore, the subsequent use of *Lupinus* should keep in mind that deficient methionine intake not only impairs the growth of cattle but also affects the metabolic pathways of sulfur. This is because the structure of methionine contains sulfur, which increases the production of lecithin in the liver by lowering cholesterol. *Bacillus*, as it has the capacity to solubilize soil nutrients, could be responsible for improving sulfur content and, therefore, improving methionine content, which explains the results observed in the present study with *B. pretiosus* [62]. Likewise, the storage and transport of calcium is of high relevance for maintaining the physiological functions of the plant, with this component having structural and signaling functions [63]. Paradoxically, a high concentration of calcium in the rhizosphere can reduce cell wall extensibility and root expansion rate, by reducing stomatal conductance, transpiration rate, and root elongation [63]. Moreover, a high concentration of calcium can disturb the lamellar membranes of the chloroplasts and disorganize the thylakoids, reducing photosynthetic efficiency and thus the net capacity for carbon assimilation throughout the plant [63]. A previous study indicates that a high calcium content in *Lupinus* can cause nutritional imbalances in some species, such as phosphorus deficiency or a reduction in magnesium, manganese, and iron concentrations affecting carbon partitioning and ultimately resulting in reduced plant growth [63].

In the present trial, the high calcium content obtained in chemical CF treatments may be an obstacle to plant growth. On the other hand, no significant differences are observed between biological treatments used with the added PGPB strains, a fact that should not be interpreted negatively. The participation of PGPBs in none of the chemical matrices favored magnesium content. However, this fact does not imply a detriment to the nutritional quality of *Lupins* since it inherently has high levels of magnesium [60, 61]. Much more relevant is the uptake of potassium in all legume crops. It participates in cellular metabolism and homeostasis contributing to cellular hydrostatic pressure (turgor), growth and responses to environmental changes [64]. Subsequently, it is necessary for a plant’s mechanical stability, nutrition, development, reproduction, and resistance to pathogens [65]. The scarcity of resources is motivating the study of how the demand for potassium from plants can be met with as little fertilizer as possible [66]. In the trial carried out, it is observed that the use of ORGAON^®^PK organic fertilizer, which contains potassium in its composition, improves the plant capture of potassium. The application of potassium can help relieve abiotic stress by increasing photosynthesized translocation and improving gas exchange, protein synthesis, enzyme activity, and stomatal conductance [65]. The use of organic fertilizers supplemented with potassium such as ORGAON^®^PK and ORGAON^®^PK-st, induces an increase in potassium concentration. This behavior is favored when you add *Pseudomonas agronomica* to either formulation of organic fertilizer. PGPB bacteria favor the assimilation of N, P, K and other plant nutrients to the crop without any chemical input to the soil, which leads to improved plant growth and an increase in crop yield [67]. They also improve the availability of nutrients in the soil by improving plant growth through the solubilization of zinc, potassium and phosphate, nitrogen fixation and the production of phytohormones [67, 68].

Between 35 and 80% of the organic matter is found in the cell wall, with the function of providing structural rigidity to the plant [69]. Depending on the composition of the cell wall, forage digestibility varies. The FDA surge (acid detergent fiber; especially cellulose and lignin) implies low food digestibility and consequently a loss of nutritional value. The low food digestibility generates a high consumption, because the FDA increases the speed of transit of the food through the digestive system, reducing the absorption of nutrients [70]. In our trial we can see that the concentrations of these molecules when biofertilizers are used do not entail a significant difference. On the other hand, the content in aFDN reaches a more abundant value when ORGAON^®^PK and ORGAON^®^PK-st are used. NDF digestibility of forage sources is often limited by cross-linking lignin to other fibrous components [71]. At the level of animal nutrition, lignin is considered an anti-nutritive component of forages since it cannot be easily fermented by lumen bacteria [72]. The result obtained from the study was that there are no significant differences within chemical treatments, maintaining between 1 and 15% of the dry mass [73]. However, the observed reduction in lignin content when *B. pretiosus* is used in the chemical matrices of ORGAON^®^PK and ORGAON^®^PK-st could favor digestibility in ruminants [74]. Lupins, with concentrations of about 7.9% starch [75] are considered poor compared to other plants, such as beans or peas [76]. On the other hand, the sugar content is relevant as it promotes the palatability of food [77]. Likewise, soluble fiber (tFDND) helps maintain a healthy gut microbiota and produce fermented compounds that are beneficial to the body, such as short-chain fatty acids, which help control cholesterol and provide a substrate for the healthy development of colon cells [78]. A higher concentration of soluble fiber in *Lupinus albus* plants could lead to an increase in the available energy, as it is limited to the concentration of fibre that is slowly and incompletely digested [79]. All these parameters are found more abundantly in plants treated with *P. agronomica. Lupinus albus* is known to produce high concentrations of polyunsaturated fatty acids [80]. Among them, linoleic acid is particularly relevant, as it is an essential fatty acid that cannot be synthesized endogenously [81]. In the present trial, fatty acid levels were higher when both PGPBs were applied in combination with CF [82]. Consistently, a previous study demonstrated that *P. agronomica* enhanced fatty acid production and promoted *Lupinus* growth [15]. Notably, the ORGAON^®^PK-st treatment supplemented with *B. pretiosus* also yielded higher fatty acid concentrations compared with its respective control, underscoring the contribution of this strain to the nutritional profile of the crop.

Despite these promising results, several limitations should be acknowledged. First, the experiment was conducted under controlled phytotron conditions, which may not fully replicate field variability. Second, the number of soil types and crop varieties tested was limited, restricting generalization. Third, functional predictions (PGPT-pred, PIFAR-pred) were based on silico analyses, which require future validation with transcriptomics and metabolomics. Addressing these limitations will be crucial to confirm the robustness and scalability of our findings.

## Conclusions

The addition of the PGPBs *Bacillus pretiosus* (C1) and *Pseudomonas agronomica* (C2) induce an increase in metabolic diversity in the rhizospheres of *Lupinus albus* in which they are added. Furthermore, both strains induce a reduction in the minimum inhibitory concentration against all antibiotics widely used in the clinic, especially amoxicillin and piperacillin (beta-lactams) and gentamicin (aminoglycoside), which is interpreted as a possible protective effect against the spread of antibiotic resistance to the environment, livestock and subsequent food chain. Moreover, the PGPB added in the biofertilizer contributes to the organic matrix in the improvement of the plant in terms of biometrics and nutritional composition. This synergistic effect is significantly greater when found in the ORGAON^®^PK and ORGAON^®^PK-st matrix. Metagenomic analyses show the survival of the strains in the rhizosphere of *Lupinus albus* during treatments. This fact may explain both the changes observed in soil microbial communities and the benefits in the nutritional quality and biometrics of the plants that host them. These results pave the way for the implications and benefits of the application of biofertilizers in pursuit of achieving a path of improvement in the quality of the plant composition as well as an increase in the production of biomass and in the practice of a more sustainable agriculture. Future research should validate these findings under field conditions, explore long-term ecological impacts on different soil types and crops, and integrate omics approaches (metatranscriptomics, metabolomics) to deepen the mechanistic understanding of how PGPBs reduce antibiotic resistance and enhance plant productivity.

## Supplementary Information

Below is the link to the electronic supplementary material.

Supplementary Material 1

## Data Availability

Sequences are available at NCBI public repository under the accession number SAMN 40643627 y PRJNA 1092974.
