# Supplementary material for: Effect of PGPB-enriched organic fertilizer ORGAON®PK on the rhizospheric microbiota and biomass of Lupinus albus (L.): a sustainable alternative to chemical fertilizer
Source: Environ Microbiome. 2025 Dec 7;21:6. doi: 10.1186/s40793-025-00827-x (PMC12797564; doi:10.1186/s40793-025-00827-x)
Supplement: Supplementary file 1 — Supplementary Material 1 [file 40793_2025_827_MOESM1_ESM.docx]

**Supplementary Material 1**.

**Table 1.** Physicochemical and microbiological composition of ORGAONPK® according to the parameters established in Royal Decree 506/2013.

| HEAVY METALS ACCORDING TO RD506/2013 | | | |
| --- | --- | --- | --- |
| Parameter | Result | Procedure | Technique |
| Total cadmium | 1.02 mg/kg | LAB 1-02-12 | ICP-MS |
| Copper | 3.8 mg/kg | LAB 1-02-12 | LAB 1-02-12 |
| Total nickel | 2.55 mg/kg | LAB 1-02-12 | LAB 1-02-12 |
| Total lead | 0.13 mg/kg | LAB 1-02-12 | LAB 1-02-12 |
| Total Zinc | 22.02 mg/kg | LAB 1-02-12 | LAB 1-02-12 |
| Total mercury | <0.02 mg/kg | LAB 1-02-12 | LAB 1-02-12 |
| Total chrome | 2.68 mg/kg | LAB 1-02-12 | LAB 1-02-12 |
| Chromium VI | <0.200 mg/kg | Outsourced | Spectrophotometry |
| Total Nitrogen | 0,5 % | LAB 1-03-70 | Volumetry |
| Water-soluble phosphorus (%P205) | 5,29 % | LAB 1-02-12 | ICP-MS |
| Water-soluble potassium (K2O) | 7,84 % | LAB 1-02-12 | ICP-MS |
| Total organic matter | 9,59 % | LAB 1-03-37 | Gravimetry |
| Total Organic Carbon | 5,56 % | LAB 1-03-37 | Gravimetry |
| pH | 5.1 pc. pH | LAB 1-03-77 | Electrometry |
| Density at 20ºC | 1.18 g/cm3 | LAB 1-03-25 | Densimetry |
| FULVIC AND HUMIC ACIDS | | | |
| Humic acids | <0.75% | LAB 1-03-45 |  |
| Fulvic acids | 6,79 % | LAB 1-03-45 |  |
| Total humic extract | 6,79% | LAB 1-03-45 |  |
| MICROBIOLOGICAL PARAMETERS | | | |
| Detection of *Salmonella* spp | Not detected at 25g | Outsourced | Enrichment and plate seeding |
| *Escherichia* *coli* count | <3.0 MPN/g | Outsourced | Most likely number |
| PARAMETER | | | |
| Furfural (2-furaldehide) | <0.03% (w/w) | Outsourced | HPLC-UV |

**Supplementary Material 2**.

**Table 1**. Soil characterization: analysis of soil aspects, physicochemical properties and particle size distribution.

| Element | Value |  | Element | ppm |
| --- | --- | --- | --- | --- |
| pH (water) | 7.1 ± 0.03 |  | AC | 3,714 ± 7.0 |
| pH (CaCl2) | 6.9 ± 0.01 |  | K | 163 ± 3.2 |
| Density (Kg/m3) | 1.15 ± 0.71 |  | Mg | 261 ± 7.3 |
| Elwctric conductivity (mmhos/cm) | 0.3 ± 0.01 |  | AI | 505 ± 5.0 |
| Organic material (%) | 1.7 ± 0.2 |  | Zn | 1.4 ± 0.8 |
| P (Bray, ppm) | 27 ± 0.9 |  | Cu | 1.89 ± 0.8 |
| P (Olsen, ppm) | 13.21 ± 2.13 |  | B | 0.5 ± 0.1 |
| S (ppm) | 3.1 ± 0.8 |  | Mn | 23 ± 1.2 |
| Texture | 2 ± 0.7 |  | Faith | 79.6 ± 2.3 |
| % Saturation Al | 0.01 ± 0.005 |  |  |  |
| Interchangeable Al (meq/100g) | 0.0022 ± 0.0002 |  |  |  |
| Sum of bases (meq/100g) | 22 ± 1 |  |  |  |
| Cation exchange capacity (meq/100g) | 21.87 ± 3.21 |  |  |  |
| %Ca | 87.90 ± 2.72 |  |  |  |
| %Mg | 10.10 ± 1.19 |  |  |  |
| %K | 2 ± 0.6 |  |  |  |

**Supplementary Material 3**.

**Table 1.** MIC values (µg. mL⁻¹) of the microbial community (cenoantibiogram) for each chemical and biological treatment. AML: amoxicillin; AUG: amoxicillin/clavulanic acid; CTX: cefotaxime; PP: piperacillin; PM: cefepime; TZP: piperacillin/tazobactam; IMI: imipenem; TS: trimethoprim/sulfamethoxazole (co-trimoxazole); IMD: imipenem + EDTA; CN: gentamicin; NA: nalidixic acid; CIP: ciprofloxacin. W: irrigation with water; CF: irrigation with chemical fertiliser; OPK: irrigation with organic fertiliser ORGAON®PK.

**Tabla 2.** Par-wise comparison (Duncan's test) against (a) beta-lactam antibiotics and (b) other antibiotic families. The mean values per treatment (n=3) are presented together with the standard deviation (SD; expressed as ±). Identical letters indicate that the mean values do not differ significantly (p < 0.05) for the same antibiotic. MIC values that have shown a statistically significant reduction with respect to their matrix control (without inoculum) are highlighted in bold. The treatments are coded as follows: C0, inoculum-free control; C1, Bacillus pretiosus; C2, Pseudomonas agronomica; W, watering with water; CF, irrigation with chemical fertilizer; OPK, irrigation with ORGAON®PK. Antibiotics used: AML (amoxicillin), AUG (amoxicillin/clavulanic acid), CTX (cefotaxime), PP (piperacillin), PM (cefepime), TZP (piperacillin/tazobactam), IMI (imipenem), TS (trimethoprim), IMD (imipenem/EDTA), CN (gentamicin), NA (nalidixic acid), CIP (ciprofloxacin).

| **a)** |  |  |  |  |  |  |  |  |  |  |  |  |
| --- | --- | --- | --- | --- | --- | --- | --- | --- | --- | --- | --- | --- |
|  |  |  |  |  |  |  |  |  |  |  |  |  |
|  | WC0 | WC1 | WC2 | CFC0 | CFC1 | CFC2 | OPKC0 | OPKC1 | OPKC2 | OPK_STC0 | OPK_STC1 | OPK_STC2 |
| AML (10E-2) | 2.56±0.00d | **0.53±0.18b** | 2.56±0.00d | 2.56±0.00d | **0.53±0.18b** | 2.56±0.00d | 1.28±0.00c | **1.17±0.18b** | 2.56±0.00d | 2.56±0.00d | **0.32±0.00a** | 2.56±0.00d |
| AUG (10E-2) | 1.71±0.74e | **0.11±0.05a** | **0.53±0.18abc** | 1.28±0.00de | **0.11±0.05a** | **0.53±0.18abc** | 1.28±0.00de | **0.27±0.00a** | **0.64±0.00bc** | 0.85±0.37cd | **0.24±0.14ab** | **0.45±0.32abc** |
| CTX (10E-2) | 0.08±0.18th | 0.01±0.00a | 0.01±0.09a | 0.11±0.08b | **0.01±0.05a** | **0.01±0.00a** | 0.12±0.38b | **0.04±0.09a** | **0.03±0.18a** | 0.05±0.18a | 0.01±0.05a | 0.02±0.03a |
| PP (10E-2) | 0.08±0.00bc | **0.01±0.01a** | **0.01±0.00a** | 0.11±0.05b | **0.01±0.00a** | **0.0±0.01a** | 0.12±0.07b | **0.04±0.01a** | **0.03±0.04a** | 0.05±0.02ab | **0.01±0.00a** | 0.02±0.01a |
| PM (10E-2) | 0.16±0.00c | **0.02±0.01a** | **0.03±0.02ab** | 0.13±0.05c | **0.01±0.01a** | **0.03±0.02ab** | 0.21±0.09d | **0.02±0.01a** | **0.03±0.01ab** | 0.08±0.00b | **0.01±0.01a** | **0.03±0.01a** |
| TZP | 4.00±0.00bcd | **1.00±0.00A** | 3.33±0.15ab | 4.67±0.06cd | **3.00±0.73a** | **1.33±0.58a** | 3.67±0.79d | **0.88±0.21abc** | **0.88±0.21abc** | 1.67±0.58d | **0.76±0.21a** | **0.77±0.39abc** |
| IMI | 8.00±0.00C | **0.35±0.28a** | **2.00±1.73a** | 4.00±0.00b | **0.76±0.21a** | **2.33±1.53a** | 8.00±0.00b | **1.67±0.58a** | **3.33±1.15a** | 3.33±1.15b | **0.76±0.21a** | **0.88±0.21a** |
|  |  |  |  |  |  |  |  |  |  |  |  |  |
| **b)** |  |  |  |  |  |  |  |  |  |  |  |  |
|  |  |  |  |  |  |  |  |  |  |  |  |  |
|  | WC0 | WC1 | WC2 | CFC0 | CFC1 | CFC2 | OPKC0 | OPKC1 | OPKC2 | OPK_STC0 | OPK_STC1 | OPK_STC2 |
| TS (10E-2) | 0.27±0.09c | **0.01±0.00a** | **0.01±0.00a** | 0.27±0.09c | **0.03±0.01a** | **0.03±0.01a** | 0.27±0.09c | **0.02±0.02a** | **0.03±0.02a** | 0.13±0.05b | **0.01±0.01a** | **0.02±0.02a** |
| IMD | 0.88±0.21c | **0.53±0.18b** | **0.42±0.18ab** | 1.67±0.58d | **0.70±0.13bc** | 1.33±0.58d | 3.00±1.73d | **0.32±0.18a** | **0.65±0.34** | 1.33±0.58d | **0.53±0.18b** | **0.43±0.18a** |
| CN | 0.50±0.00ab | **0.33±0.14c** | **0.29±0.19de** | 0.83±0.29ab | **0.42±0.14cd** | **0.33±0.14ef** | 1.33±0.58abc | **0.25±0.07f** | **0.29±0.19f** | 1.00±0.00A | **0.17±0.07d** | **0.17±0.07d** |
| NA (10E-2) | 2.56±0.00c | **0.59±0.61ab** | **0.85±0.37ab** | 2.56±0.00c | **0.85±0.37ab** | **1.28±1.11b** | 2.56±0.00c | **0.75±0.49ab** | **0.85±0.37ab** | 2.56±0.00c | **0.48±0.28a** | **0.53±0.18ab** |
| CIP | 0.83±0.29e | **0.50±0.00bcd** | **0.50±0.0bcd** | 1.33±0.58e | **0.58±0.38cd** | **0.29±0.19bcd** | 1.33±0.19e | **0.21±0.00d** | **0.25±0.00bcd** | 0.67±0.29e | **0.13±0.00abc** | **0.17±0.07ab** |
|  |  |  |  |  |  |  |  |  |  |  |  |  |

**Supplementary Material 4**.

**Table 1**: Effect of fertilizers and bacteria on soil microbial community structure (ADONIS analysis). **Df** is the degrees of freedom; **Sums Of Sqs** is the sum of squares; **MeanSqs** is the average of the mean squares; **F** indicates the relationship between variability explained by variables compared to unexplained variability; **R2** is the proportion of variability explained by the model; and **Pr**(>F) is the p-value, which indicates the statistical significance of the model. The ' **NaN**' values indicate that they are not applied for that row or are not available.

| **Variable** | **Mexico City** | **Sums Of Sqs** | **MeanSqs** | **F. Model** | **R2** | **Pr(>F)** |
| --- | --- | --- | --- | --- | --- | --- |
| Fertilizer | 2 | 0,008631 | 0,004315 | 0,861372 | 0,166475 | 0,615 |
| Bacterium | 2 | 0,008144 | 0,004072 | 0,812825 | 0,157092 | 0,687 |
| Residuals | 7 | 0,035070 | 0,005010 | Nan | 0,676434 | Nan |
| Total | 11 | 0,051845 | Nan | Nan | 1,000000 | Nan |

**Supplementary Material 5.**

**Table 1.** Analysis of the Alpha Diversity Index of the Soil Microbial Community in Response to Treatments.

| CF - ORGAON PK | H | p-value | q-value |
| --- | --- | --- | --- |
| Faiths statistics | 5 | 0.02 | 0.07 |
| Evenness statistics | 0.02 | 0.88 | 0.88 |
| Shannon Index | 0.06 | 0.79 | 0.82 |
| Observed features | 0 | 1 | 1 |
| CF - WATER | H | p-value | q-value |
| Faiths statistics | 0.42 | 0.51 | 0.51 |
| Evenness statistics | 1.19 | 0.27 | 0.41 |
| Shannon Index | 0.04 | 0.82 | 0.82 |
| Observed features | 0.04 | 0.82 | 1 |
| ORGAON PK - WATER | H | p-value | q-value |
| Faiths statistics | 0.55 | 0.45 | 0.51 |
| Evenness statistics | 1.80 | 0.17 | 0.41 |
| Shannon Index | 0.06 | 0.79 | 0.82 |
| Observed features | 0.06 | 0.79 | 1 |

**Table 2.** Analysis of the Alpha Diversity Index of the Soil Microbial Community in Response to the Inoculated Strains.

| C1-C2 | H | p-value | q-value |
| --- | --- | --- | --- |
| Faiths statistics | 1.25 | 0.72 | 0.72 |
| Evenness statistics | 1.25 | 0.72 | 0.72 |
| Shannon Index | 0.08 | 0.77 | 1 |
| Observed features | 0.08 | 0.77 | 1 |
| C1-Control | H | p-value | q-value |
| Faiths statistics | 1.25 | 0.72 | 0.72 |
| Evenness statistics | 0.49 | 0.47 | 0.71 |
| Shannon Index | 0 | 1 | 1 |
| Observed features | 0 | 1 | 1 |
| C2-Control | H | p-value | q-value |
| Faiths statistics | 0.33 | 0.56 | 0.72 |
| Evenness statistics | 0.75 | 0.38 | 0.71 |
| Shannon Index | 0.08 | 0.77 | 1 |
| Observed features | 0.08 | 0.77 | 1 |

**Figure 1.** Beta Diversity Analysis of the Taxonomic Structure of Soil Microorganisms Using Principal Coordinates with Weighted Unifrac Metric.


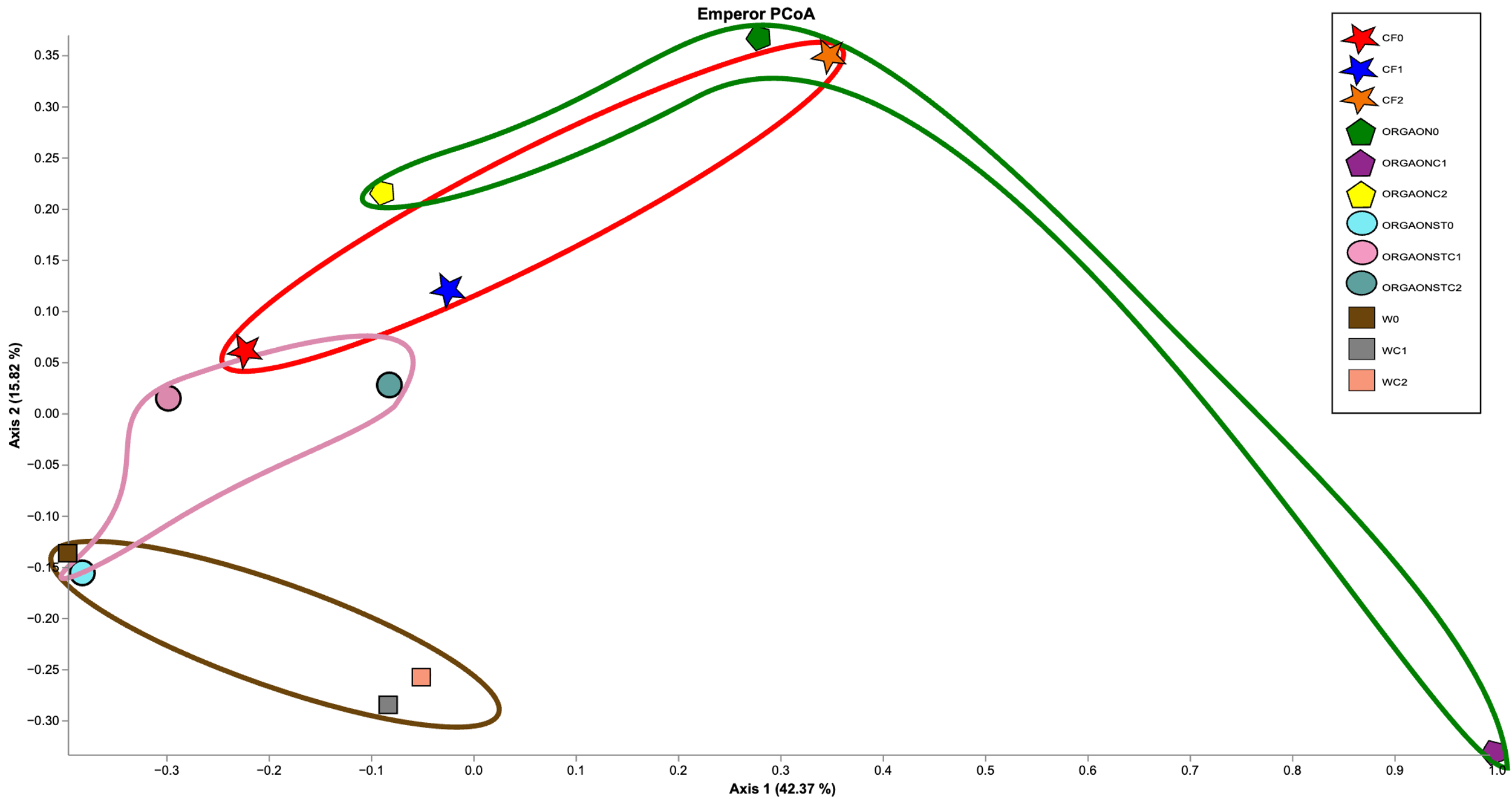


**Figure 2.** Analysis of Microbial Composition Among Treatment Groups and Effects of Strain Addition on the Microbiota.


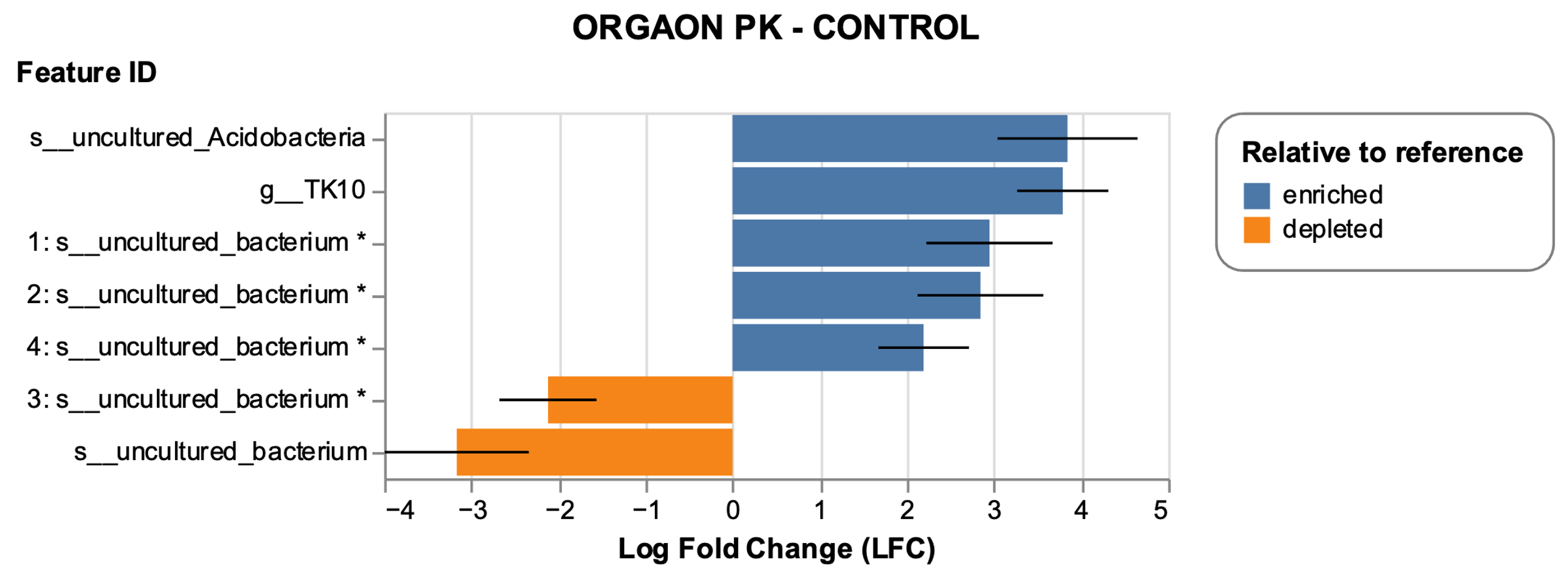

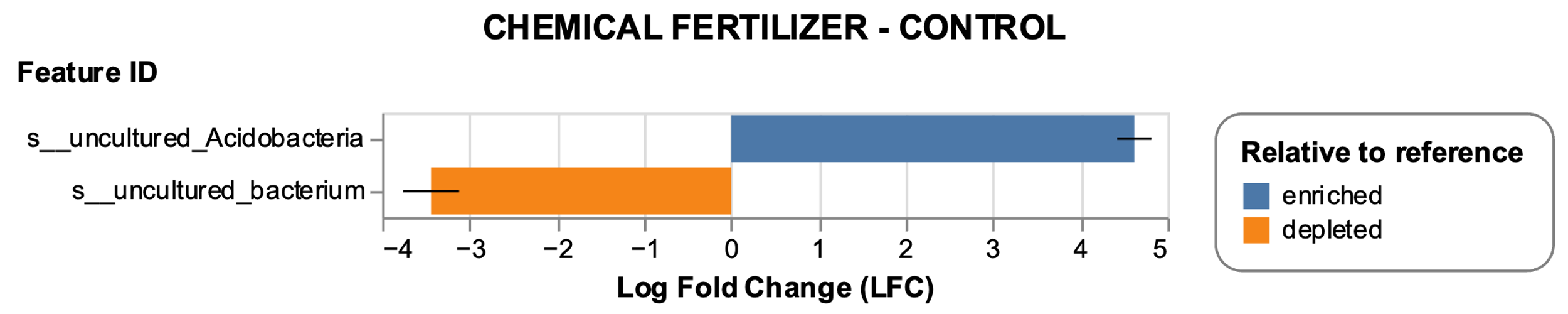

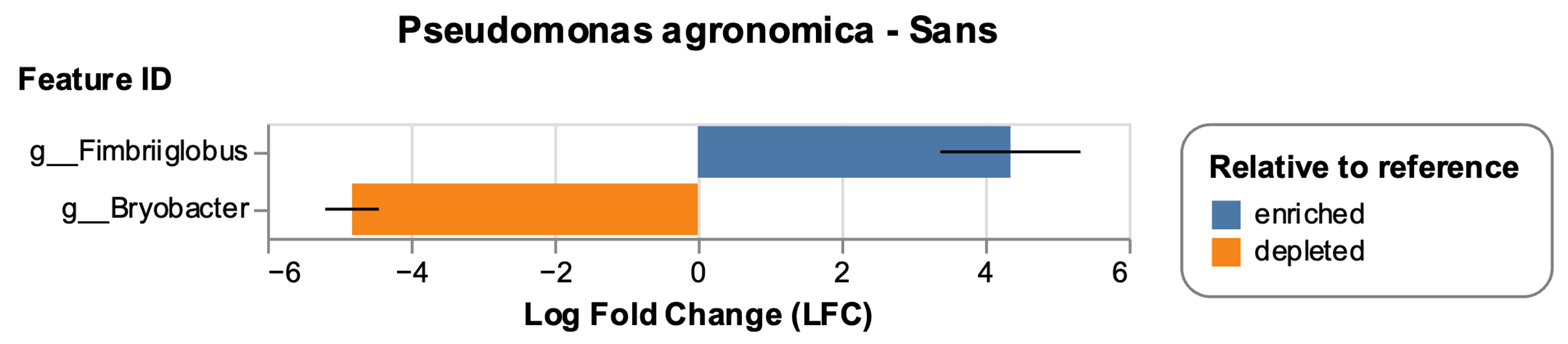

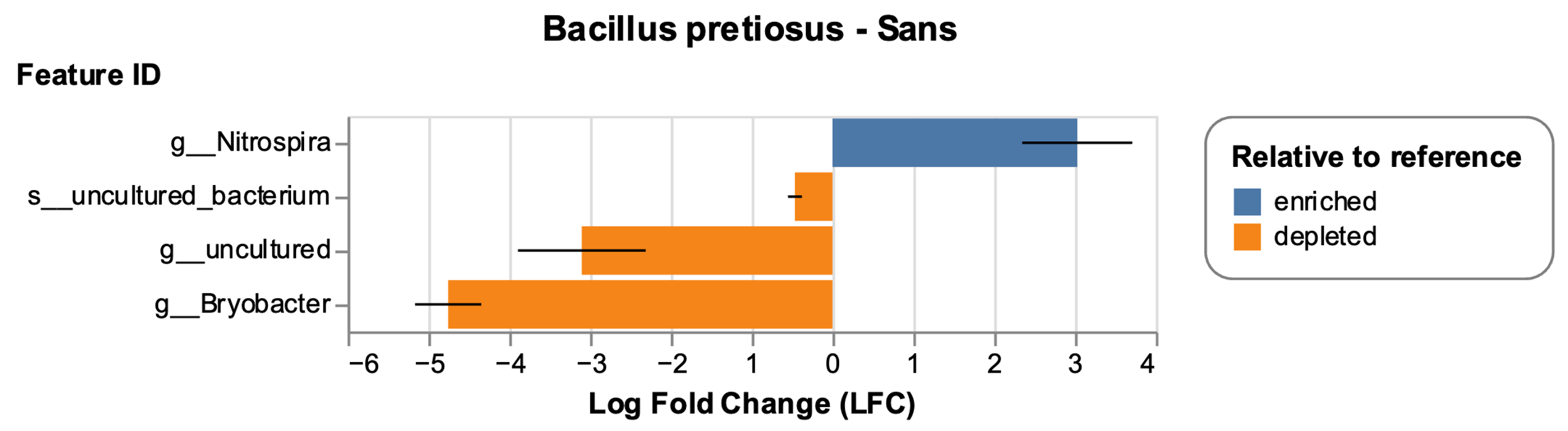


**Supplementary Material 6.**

**Table 1. (A)** Nutritional data related to crude protein content. Mean values for n = 3. Bars with identical letters indicate that mean values are not significantly different (p-value < 0.05). **(B)** Nutritional data related to carbohydrate (sugar) content. Mean values for n = 3. Bars with identical letters indicate that mean values are not significantly different (p-value < 0.05). Letter coding: [a-d] represents mean comparisons for lignin content (%DM); [h-k] for starch content (%DM); [w-z] for water-soluble carbohydrate (WSC, %DM). %DM (percentage of dry matter). **(C)** Nutritional data related to methionine amino acid content. Mean values for n = 3. Bars with identical letters indicate that mean values are not significantly different (p-value < 0.05). Letter coding: [a-e] represents mean comparisons for crude protein; [x-z] for soluble protein. %DM (percentage of dry matter). **(D)** Nutritional data related to carbohydrate (digestible fibre) content. Mean values for n = 3. Bars with identical letters indicate that mean values are not significantly different (p-value < 0.05). Letter coding: [a-d] represents mean comparisons for acid detergent fibre (ADF); [w-z] for amylase-treated neutral detergent fibre (aNDF). %DM (percentage of dry matter). **(E)** Nutritional data related to fibre digestibility. Mean values for n = 3. Bars with identical letters indicate that mean values are not significantly different (p-value < 0.05). Letter coding: [a-e] represents mean comparisons for total tract digestible fibre (tFDND). **(F)** Nutritional data related to fatty acid (FA) content. Mean values for n = 3. Bars with identical letters indicate that mean values are not significantly different (p-value < 0.001). **(G)** Nutritional data related to mineral content. Mean values for n = 3. Bars with identical letters indicate that mean values are not significantly different (p-value < 0.05). Letter coding: [a-f] represents mean comparisons for calcium content; [h-k] for magnesium content; [m-t] for potassium content (x/10); [x-z] for sulphur content. %DM (percentage of dry matter).

| 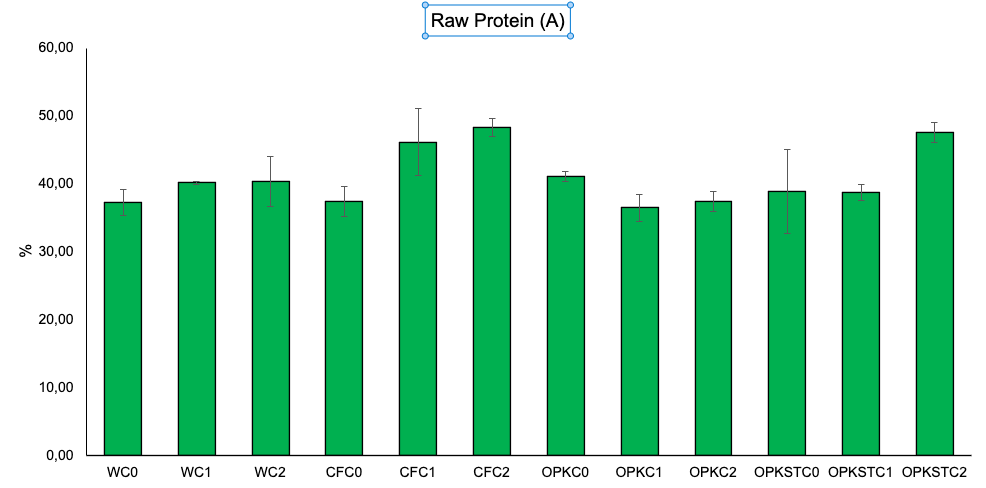  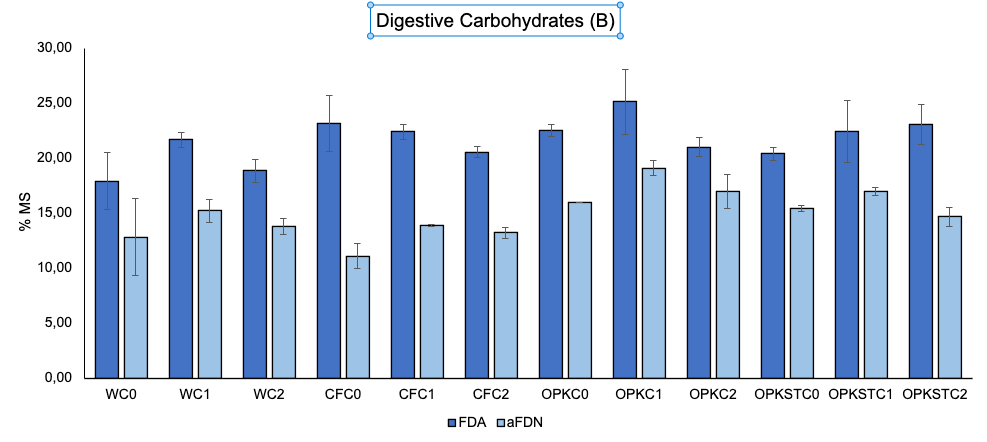  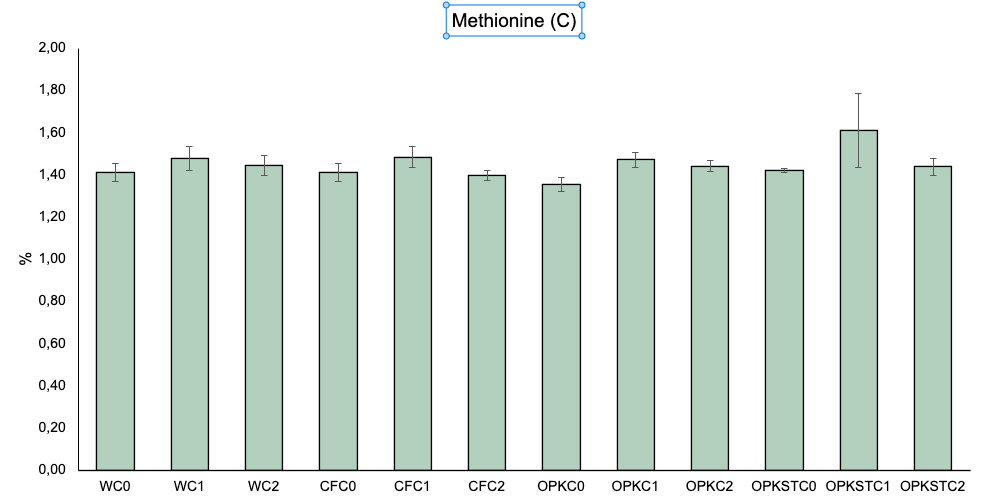  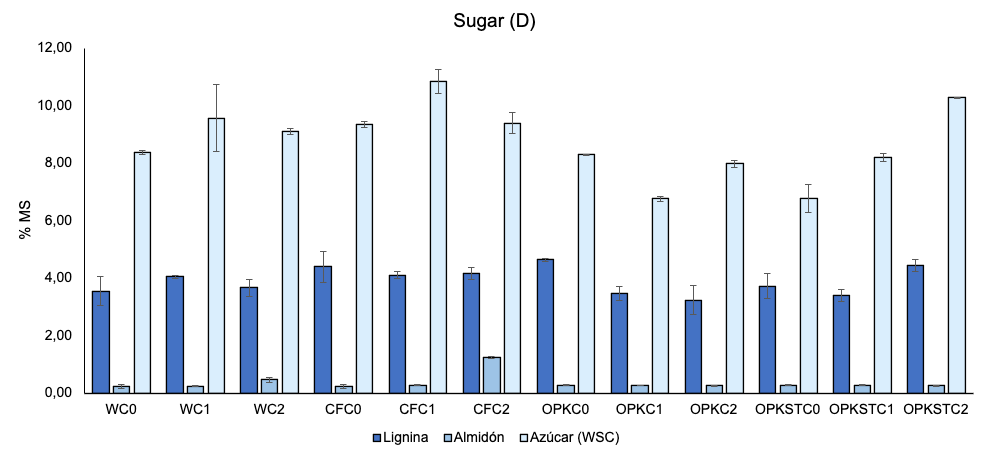  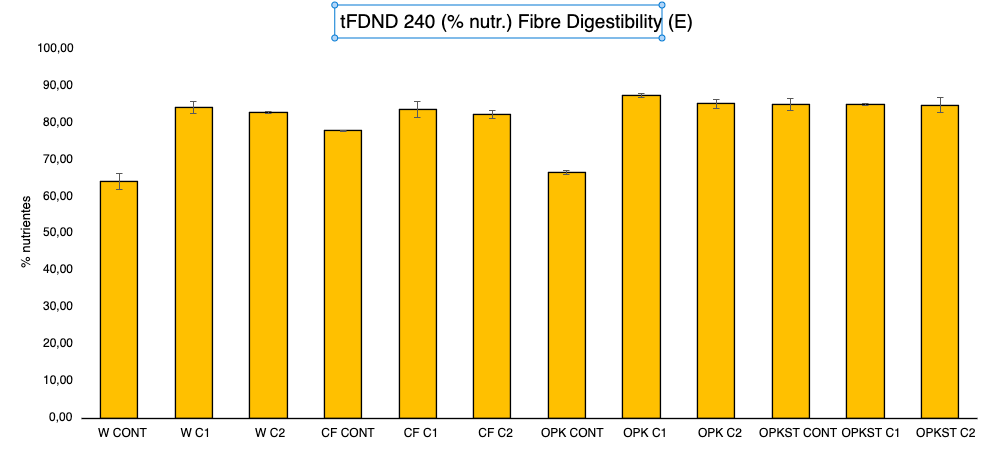  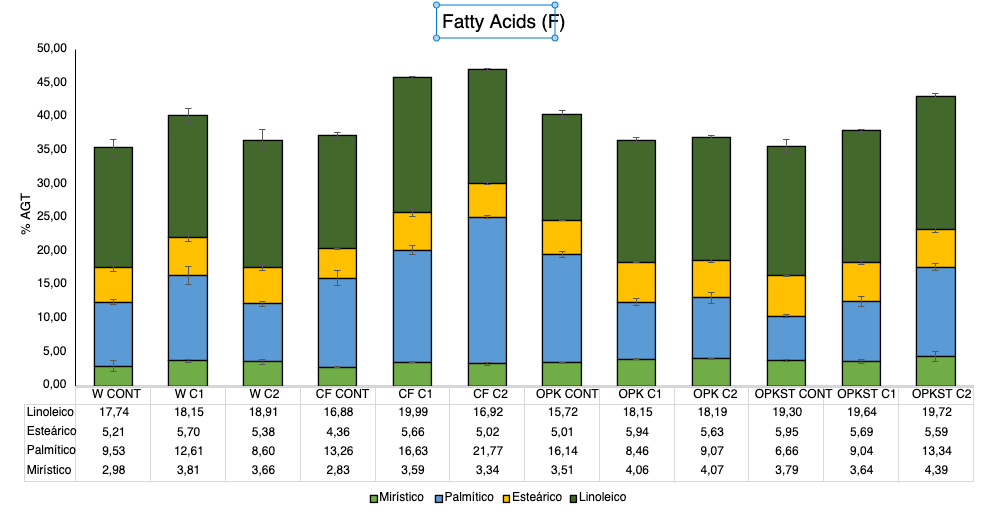  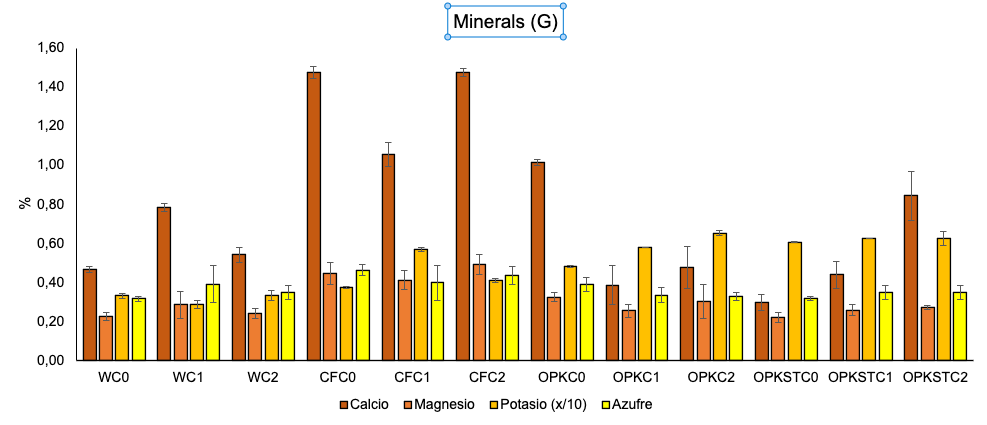 |
| --- |
